# Excessive accumulation of epicardial adipose tissue promotes microvascular obstruction formation after myocardial ischemia/reperfusion through modulating macrophages polarization

**DOI:** 10.1186/s12933-024-02342-8

**Published:** 2024-07-05

**Authors:** Jinxuan Zhao, Wei Cheng, Yang Dai, Yao Li, Yuting Feng, Ying Tan, Qiucang Xue, Xue Bao, Xuan Sun, Lina Kang, Dan Mu, Biao Xu

**Affiliations:** 1 Department of Cardiology, MOE Key Laboratory of Model Animal for Disease Study, Nanjing Drum Tower Hospital, The Affiliated Hospital of Nanjing University Medical School, Nanjing University, Nanjing, China; 2 Division of Colorectal Surgery, Department of General Surgery, Nanjing Drum Tower Hospital, The Affiliated Hospital of Nanjing University Medical School, Nanjing University, Nanjing, China; 3 Department of Cardiology, Nanjing Drum Tower Hospital, Clinical College of Nanjing Medical University, Nanjing, China; 4 Department of Radiology, Nanjing Drum Tower Hospital, The Affiliated Hospital of Nanjing University Medical School, Nanjing University, Nanjing, China

**Keywords:** Epicardial adipose tissue, Microvascular obstruction, Inflammation, Macrophage, Liraglutide, GLP-1/GLP-2 receptor dual agonist

## Abstract

**Background:**

Owing to its unique location and multifaceted metabolic functions, epicardial adipose tissue (EAT) is gradually emerging as a new metabolic target for coronary artery disease risk stratification. Microvascular obstruction (MVO) has been recognized as an independent risk factor for unfavorable prognosis in acute myocardial infarction patients. However, the concrete role of EAT in the pathogenesis of MVO formation in individuals with ST-segment elevation myocardial infarction (STEMI) remains unclear. The objective of the study is to evaluate the correlation between EAT accumulation and MVO formation measured by cardiac magnetic resonance (CMR) in STEMI patients and clarify the underlying mechanisms involved in this relationship.

**Methods:**

Firstly, we utilized CMR technique to explore the association of EAT distribution and quantity with MVO formation in patients with STEMI. Then we utilized a mouse model with EAT depletion to explore how EAT affected MVO formation under the circumstances of myocardial ischemia/reperfusion (I/R) injury. We further investigated the immunomodulatory effect of EAT on macrophages through co-culture experiments. Finally, we searched for new therapeutic strategies targeting EAT to prevent MVO formation.

**Results:**

The increase of left atrioventricular EAT mass index was independently associated with MVO formation. We also found that increased circulating levels of DPP4 and high DPP4 activity seemed to be associated with EAT increase. EAT accumulation acted as a pro-inflammatory mediator boosting the transition of macrophages towards inflammatory phenotype in myocardial I/R injury through secreting inflammatory EVs. Furthermore, our study declared the potential therapeutic effects of GLP-1 receptor agonist and GLP-1/GLP-2 receptor dual agonist for MVO prevention were at least partially ascribed to its impact on EAT modulation.

**Conclusions:**

Our work for the first time demonstrated that excessive accumulation of EAT promoted MVO formation by promoting the polarization state of cardiac macrophages towards an inflammatory phenotype. Furthermore, this study identified a very promising therapeutic strategy, GLP-1/GLP-2 receptor dual agonist, targeting EAT for MVO prevention following myocardial I/R injury.

**Supplementary Information:**

The online version contains supplementary material available at 10.1186/s12933-024-02342-8.

## Background

Epicardial adipose tissue (EAT), originating from the splanchnopleuric mesoderm, refers to a metabolically active fat depot lies between the myocardium and visceral pericardium [1]. Due to its distinctive distribution, paracrine effects and metabolic properties, EAT has gained extensive attention as a promising imaging biomarker of metabolic diseases and cardiovascular diseases [2]. High amount of EAT accumulation has been associated with the initiation and development of diabetes [3, 4], atherosclerosis [5], coronary artery disease [6, 7] and atrial fibrillation [8]. Although a relation between EAT volume and the severity of acute myocardial infarction (AMI) has been reported recently, the exact role of EAT in the development of myocardial ischemia/reperfusion (I/R) is still controversial and the underlying mechanisms involved in this relationship have not been fully elucidated [9–11].

Microvascular obstruction (MVO), occurs in about 50% of ST-elevation myocardial infarction (STEMI) following reperfusion treatment, has been recognized as an independent risk factor for poor prognosis in AMI patients [12]. Numerous clinical trials have demonstrated that patients with the presence of MVO following primary percutaneous coronary intervention (pPCI) suffer from adverse left ventricular remodeling, poor cardiac function and high mortality rates [13–15]. There has been growing recognition that further insight into MVO formation post-reperfusion is critical for identifying the therapeutic targets to minimize the reperfusion injury [16]. Unfortunately, which kind of patients are susceptible to MVO remains elusive. Growing evidence indicated that microvascular dysfunction and inflammation over-activation were two major mechanisms of MVO formation [12, 17]. EAT embeds the coronary arteries and shares microcirculation with the underlying myocardium. Considering its close proximity to the coronary arteries, it is possible that EAT exerts profound effect on myocardial microvascular function and thereby influences the progression of MVO formation. Additionally, as an active endocrine organ, EAT secretes numerous pro-inflammatory and anti-inflammatory adipocytokines [18–21], suggesting its potential role in cardiac inflammation modulation. Thus, it is supposed that EAT might be involved in the process of MVO formation via both paracrine and vasocrine mechanisms.

Cardiac magnetic resonance (CMR) has been recognized as the gold standard for the measurements of cardiac function, infarct size and MVO [22]. CMR is also a valuable tool for identifying the distribution and volume of EAT with high sensitivity and specificity, which provides more accurate quantification of EAT than Computed Tomography (CT) and echocardiography [23, 24]. Furthermore, CMR has the advantage that it allows for both measurements in a single examination. The aim of the study was to evaluate the predictive capacity of the distribution and amount of EAT for MVO formation as measured by CMR in individuals with STEMI following pPCI and explored the underlying mechanisms in a mouse model. Our study unraveled the pro-inflammatory effect of EAT in MVO formation under myocardial I/R condition and provided novel therapeutic insight in EAT-targeted therapeutic strategies to minimize I/R injury in the future.

## Methods

### Study population

STEMI patients who were admitted to Nanjing Drum Tower Hospital, Medical School of Nanjing University from July 2018 to September 2019 were included in this retrospective study. Based on the current European Guidelines, STEMI patients underwent pPCI successfully [25]. We excluded patients with any of the following characteristics: older patients (> 85 years), A history of myocardial infarction (MI), renal failure with estimate glomerular filtration < 30 ml/min per 1.73 m^2^, cardiac shock, sustained ventricular tachycardia and ventricular fibrillation after STEMI, received GLP-1 agonists, GLP-2 agonists or DPP4 inhibitors treatment 6 months before or during hospitalization as well as contraindications to CMR. After exclusion, a total of 134 patients were enrolled. Finally, 129 STEMI patients were included in the study after excluding 5 patients due to poor image quality of CMR (Fig. S1). The study was approved by the Institutional Ethics Committee of Nanjing Drum Tower Hospital (Approval No. 202153101) according to Helsinki declaration. Written informed consent was obtained from each patient upon enrollment.

Demographic information, medical history of traditional cardiovascular risk factors, vital parameters such as heart rate and blood pressure, Killip class and current cardiovascular medications were collected. Physicians performed the basic physical examinations for each patients. Body mass index (BMI) was calculated by dividing weight (kg) by height (m^2^). Body surface area (BSA) was calculated using the DuBois and DuBois formula.

### Blood sample measurement

Upon admission, biochemistry data such as Troponin T (TnT) and Brain natriuretic peptide (BNP) were assessed, with follow-up evaluations at 6 h post pPCI and daily until the patient’s discharge. Routine blood tests including fasting glucose, glycosylated hemoglobin, creatinine, lipid profiles, and C-reactive protein (CRP) were conducted at Nanjing Drum Tower Hospital’s central laboratory following an overnight fast using standard laboratory methods at day 1 after pPCI. Blood samples for specialized tests were collected at a fasting state at day 1 after pPCI. Following centrifugation at 1200 g for 15 min, plasma samples were collected and immediately stored at 80 °C until further analysis. Total Glucagon-like peptide 1 (GLP-1), Glucagon-like peptide 2 (GLP-2) and Dipeptidyl peptidase-4 (DPP4) levels in plasma were determined by Total GLP-1 enzyme-linked immunosorbent assay (ELISA) Kit (Millipore Corporation, Billerica, MA), Total GLP-2 ELISA Kit (Merck S.p.A, Milan, Italy) and DPP4 ELISA Kit (Cusabio, Wuhan, China), respectively. ELISA tests were conducted in accordance with the instructions provided by the manufacturers. As described previously, we measured the cleavage rate of 7-amino-4-methylcoumarin (AMC) from the synthetic substrate H-glycyl-prolyl AMC (Sigma Aldrich, USA) to determine DPP4 activity [26, 27]. The fluorescence of the AMC (excitation/emission-380/460 nm) was measured on a plate reader (Tecan).

### Coronary angiography analysis

All coronary angiograms were analyzed by two experienced interventional cardiologists who were blinded to the CMR information of the patients. The following parameters were collected: infarct-related artery, stent parameters, first medical contact (FMC) to wire-crossing time and Thrombolysis In Myocardial Infarction (TIMI) flow grade before and after pPCI. The complexity and severity of coronary atherosclerotic lesion were defined using the Syntax score, a lesion-based angiographic scoring system which was calculated using the Syntax score algorithm available at www.syntaxscore.com.

### CMR imaging protocol

Each patient received the CMR imaging scan using a 3.0-T MRI system (Ingenia, Philips, Netherlands) approximately 4.9 ± 1.7 days after pPCI. All images were obtained during breath-holding at end expiration using vector cardiograph gating with the patients in supine position. The scan protocol was performed in accordance with the guidelines of the Society of Cardiovascular Magnetic Resonance [28]. Initially, the traditional multi-position spin-echo (SE) sequence scan was performed. Then, the balanced turbo field echo (B-TFE) cine sequence was scanned for each patient, including two-chamber long-axis views, three-chamber long-axis views, four-chamber long-axis views as well as continuous stacks of short-axis images covering the entire left ventricle [parameters: time of repetition (TR): 2.9 ms; time of echo (TE): 1.47 ms; flip angle (FA): 45°; slice thickness: 8 mm]. For late gadolinium enhancement (LGE) imaging, an infusion of 0.3 mmol/kg gadodiamide contrast agent was given (GE Pharmacy, Shanghai, China; 0.3 mmol/kg; velocity: 2.0 m/s). Ten minutes later, the long-axis (four-chamber) and short-axis images of the whole left ventricle were acquired using an inversion recovery gradient echo sequence (parameters: flip angle 25°; TR/TE 6/3 m; TI 260–350 ms; voxel 1.6 × 1.9 × 8 mm^3^; shot duration 100–125 ms).

### CMR analysis

**Fig. 1:**
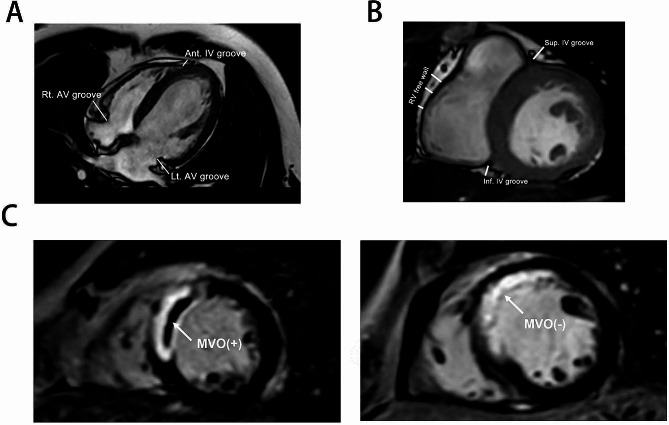
Representative images of cardiac magnetic resonance of STEMI patients. **A** Measurements of EAT thickness in the grooved segments at three different locations in the horizontal long-axis plane (left AV groove, right AV groove and anterior IV groove). **B** Measurements of EAT thickness in the grooved segments at three locations in the short-axis plane (superior IV groove, inferior IV groove and the right ventricular free wall. **C** Short axis LGE sequence performed at mid left ventricle level showing the presence of MVO

The CMR images were interpreted independently by 2 experienced radiologists blinded to the angiographic and clinical information of the patients using Q-MASS MR 8.1 (Medis, Leiden, The Netherlands) [10]. Left ventricular end-systolic volume index (LVESVI), left ventricular end-diastolic volume index (LVEDVI) and left ventricular ejection fraction (LVEF%) were measured by outlining the left ventricular (LV) endocardial and epicardial borders on the short-axis images at end-diastole and end-systole phases. The full-width at half-maximum method (FWHM) was applied to delineate the infarct core in LGE images. A hypoenhanced region within the hyperenhanced area in LGE images was defined as MVO [22]. The fat located between the outer margin of myocardium and the visceral pericardium was defined as EAT. As shown in Fig. 1, EAT thickness in the grooved segments was measured at three separate sites in the horizontal long-axis image (left atrioventricular groove, right atrioventricular groove and anterior interventricular groove) and two sites in the basal short-axis image (inferior interventricular groove and superior interventricular groove and) (Fig. 1B). We measured the EAT thickness over right ventricular free wall at three equally spaced points in the basal short-axis image along the right ventricular free wall and used the mean of the three measurements in the statistical analysis [29]. EAT representative cross-sectional areas were measured on the standardized ventricular short-axis planes at the basal, midcavity, and apical levels as well as the horizontal long-axis plane. Measurements of EAT thickness and cross-sectional area were conducted at the endo-diastolic phase. For epicardial fat volume determination, EAT area was captured from consecutive short-axis images at end-diastole phase by manual tracing and multiplied by the slice thickness to yield the fat volume. Partial volumes were obtained by multiplying the segmented cross-sectional areas by the section thickness. Total epicardial fat volume and total left atrioventricular epicardial fat volume (LV EAT) were obtained after the summation of data of all partial volumes. To determine the mass of EAT, the volume was multiplied by 0.9196 g/cm^3^, as described in a previous study [30]. For EAT mass index (g/m^2^) calculation, the EAT mass was divided by the BSA.

### Animal experimental protocol

Upon approval by the Institutional Ethics Committee of Nanjing Drum Tower Hospital (Approval No. 20,011,141), animal experiments were conducted in accordance with “Animal research: reporting of in vivo experiments” (ARRIVE) guidelines. 6 week old male C57BL/6J mice were purchased from Nanjing University Model Animal Research Center and housed in a temperature (22 ± 1℃) and humidity (65–70%) controlled room with a 12 h light and dark cycle. Mice were fed a high-fat diet (HFD, 60% fat, Research Diets, D12492) for 10 weeks before myocardial I/R induction. To induce myocardial I/R injury, anesthesia was achieved with 1.5 to 2% isoflurane and the mice were ventilated artificially with a rodent ventilator. Afterwards, thoracotomy was carried out and EAT was gently removal in mice randomized to I/R-EAT group, while preserved in I/R + EAT group. The left anterior descending coronary artery (LAD) was carefully exposed, ligated using a 7 − 0 silk suture and subsequently released after 60 min as previously described [17]. Animals in sham group underwent similar procedures without LAD ligation. Mice received the high fat diet for the whole experiment’s duration until they were killed. For GLP-1 receptor agonist (liraglutide) and GLP-1/GLP-2 receptor dual agonist (dapiglutide) administration, mice were randomly assigned to one of four groups: sham, vehicle, liraglutide (MedChemexpress, 300 µg/kg s.c.) or dapiglutide (MedChemexpress, 30 nmol/kg s.c.). Treatment was administered daily 5 days before myocardial I/R induction and administered every two days following operation. The doses of liraglutide and dapiglutide were determined according to previously published studies [31–33]. As for –EAT + V, -EAT + L and –EAT + D group, EAT removal was performed before treating the mice with liraglutide or dapiglutide as the above experimental design.

### Myocardial infarct size measurement

Myocardial infarction size (IS) was determined by Evans blue dye and 2,3,5-triphenyl-2 H-tetrazolium chloride (TTC) staining. In order to determine the non-perfused myocardium, 1% Evans Blue dye (Sigma Aldrich) was injected into the aorta after LAD re-ligation. Afterward, hearts were quickly frozen at − 80 °C and then sliced into 1 mm thick slices. Slices were stained with 1.5% TTC (Sigma Aldrich) for 15 min in the dark and analyzed using Image J as previously reported [34]. The Evans blue unstained areas represented area at risk (AAR). The AAR but viable tissue was stained by TTC in red, while the infracted myocardium appeared paler. Percentage AAR was expressed as AAR area/total area of myocardium and the percentage of infarct was determined by infarct area/AAR.

### Preparation of conditioned media from EAT (EAT-CM)

EAT-CM was prepared from the EAT of mice in the I/R + EAT group according to previous study [35]. After washing with saline, the EAT was cut into small fragments and cultured in DMEM-F12 medium with 10% FBS, 17 µmol/L D-pantothenate, 33 µmol/l biotin and 1% Penicillin/Streptomycin at 37 °C. On the second day, the medium were replaced with serum-free culture system as descried above. One day later, the EAT samples were removed and the EAT-CM was collected for subsequent experiments. In order to prepare the EVs-free AT conditional medium (CM), the collected culture medium was centrifuged at 110,000 g for 70 min for extracellular vesicles elimination. The supernatants were then purified through 0.45 μm filters and collected for subsequent macrophage culture. Detailed descriptions of other methods are provided in Supplementary Material.

### Statistical analysis

Continuous data were presented as mean ± standard deviation (SD) or as median and interquartile range in cases of abnormal distribution. Categorical data were shown as numbers (n) or percentages (%). Differences between the groups were tested using Student’s t-test for normally distributed variables or Mann–Whitney test for variables with skewed distributions. The multiple comparisons were performed using one-way ANOVA followed by Tukey’s multiple comparisons test. Two-way ANOVA followed by Bonferroni’s multiple comparisons test was used to determine differences between groups at multiple time points. Categorical variables were compared using Chi-square tests or Fisher’s exact test as appropriate. Linear regression analysis was performed to identify correlations between the EAT volume and the quantitative variables, such as cardiovascular risk factors, laboratory parameters and CMR parameters. The associations between parameters were assessed using Pearson’s correlation test for parametric variables or Spearman’s correlation for non-parametric variables. The associations between clinical covariates and MVO formation were evaluated using Binary logistic regression (results presented as odds ratio [OR] and 95% confidence interval [CI]). Variables with the P value less than 0.10 in univariable analysis were further included as covariates in the multivariable analysis. A receiver-operating characteristic (ROC) curve analysis was done to evaluate the accuracy of EAT volume parameters to predict MVO formation. ROC-comparisons were performed using the method suggested by DeLong et al [36]. All statistical analyses were performed using SPSS version 23.0 (IBM, New York, USA) and MedCalc version 18.2.1 (MedCalc, Belgium). A 2-tailed p-value < 0.05 was considered statistically significant.

## Results

### Clinical characteristics of patients with MVO

Based on the flow chart in Figure S1, 129 patients were enrolled in this study. STEMI Patients were subdivided into MVO (*n* = 83) and non-MVO (*n* = 46) subgroups, according to the presence or absence of MVO measured by CMR (case example shown in Fig. 1C). The baseline clinical characteristics were comparable between the two groups (Table 1). As for laboratory parameters comparisons, only neutrophil lymphocyte ratio (NLR), CRP, and peak TnT showed statistically significant differences between the two groups. The patients with MVO were found to have higher level of NLR (6.146 ± 2.295 vs. 4.544 ± 2.251, *p* < 0.001) and CRP (5.978 ± 2.534 vs. 5.072 ± 2.393, *p* = 0.0493) compared with patients without MVO, suggesting high intensity of inflammation in patients with MVO. As for angiographic characteristics, the infarct related artery, the syntax score, FMC to wire time, TIMI flow grade and the formation of collateral circulation did not differ significantly between the two groups. Furthermore, the number, diameter, and length of stents were not associated with MVO formation (Table 2). In CMR functional analysis, larger infarct size (24.55 ± 10.28 vs. 11.05 ± 8.894, *p* < 0.001), impaired LVEF% (43.31 ± 9.855 vs. 55.63 ± 6.941, *p* < 0.001), larger LVEDVI (64.80 ± 15.15 vs. 55.42 ± 14.11, *p* < 0.001) and LVESVI (37.19 ± 12.31 vs. 25.05 ± 9.021, *p* < 0.001) were found in MVO group as compared with non-MVO group (Table 3).

**Table 1:** Baseline characteristics between patients with or without MVO

Parameter	Patients without MVO, *n* = 46	Patients with MVO, *n* = 83	*P* value
Age, years	58.02 ± 11.96	61.40 ± 11.32	0.1142
Male gender, n (%)	39 (84.78%)	73 (87.95%)	0.6103
BMI (kg/m^2^)	25.19 ± 2.593	25.00 ± 3.023	0.7160
BSA (m^2^)	1.836 ± 0.1245	1.808 ± 0.1302	0.2266
Arterial hypertension, n (%)	20 (43.48%)	44 (53.01%)	0.2996
Diabetes mellitus, n (%)	12 (26.09%)	36 (43.37%)	0.0517
Smoking, n (%)	27 (58.70%)	48 (57.83%)	0.9241
Family history of CAD, n (%)	1 (2.17%)	3 (3.61%)	1.000
Hypercholesterolemia, n (%)	14 (30.43%)	25 (30.12%)	0.9703
Clinical signs and symptoms of angina before MI, n (%)	14 (30.43%)	24 (28.91%)	0.8561
Prior cerebral infarction, n (%)	2 (4.35%)	3(3.61%)	1.000
Previous stent implantation, n (%)	3 (6.52%)	3 (3.61%)	0.666
Systolic blood pressure, mm Hg	130 ± 21	127 ± 20	0.3556
Diastolic blood pressure, mm Hg	83 ± 11	83 ± 13	0.8017
Heart rate, bpm	79 ± 14	83 ± 18	0.3057
**Acute heart failure (Killip classifcation)**			
Class I, n (%)	43 (93.48%)	71 (85.54%)	0.1780
Class II, n (%)	2 (4.35%)	8 (9.64%)	0.493
Class III, n (%)	1 (2.17%)	2 (2.41%)	1.000
Class IV, n (%)	0	2 (2.41%)	0.538
**Medication after infarction**			
Aspirin, n (%)	46 (100%)	83 (100%)	1.000
Clopidogrel, n (%)	10 (21.74%)	23 (27.71%)	0.4565
Ticagrelor, n (%)	36 (78.26%)	60 (72.29%)	0.4565
Statins, n (%)	45 (97.83%)	83 (100%)	0.357
β-blocker, n (%)	32 (69.57%)	62 (74.70%)	0.5299
Nitrate, n (%)	29 (63.04%)	60 (72.29%)	0.2768
ACEI/ARB, n (%)	31 (67.39%)	56 (67.47%)	0.9927

BSA, body surface area; ACEI, angiotensin-converting enzyme; ARB, angiotensin II receptor blockers; CAD, coronary artery disease; Data are presented as mean ± SD or as number (%) affected

**Table 2:** Laboratory and angiographic characteristics between patients with or without MVO

Parameter	Patients without MVO, *n* = 46	Patients with MVO, *n* = 83	*P* value	
Hemoglobin, g/L	144.8 ± 14.21	143.7 ± 18.36	0.9990	
WBC, x10^9^/L	10.24 ± 2.525	10.44 ± 3.035	0.8861	
Neutrophil, x10^9^/L	7.472 ± 2.484	7.956 ± 2.877	0.3384	
NLR	4.544 ± 2.251	6.146 ± 2.295	< 0.001	
CRP, mmol/L	5.072 ± 2.393	5.978 ± 2.534	0.0493	
Platelet, x10^9^/L	223.2 ± 57.42	210.7 ± 80.17	0.1030	
Total cholesterol, mmolL	4.638 ± 1.012	4.565 ± 1.066	0.7623	
LDL-C, mmol/L	2.989 ± 0.945	2.773 ± 0.917	0.2071	
Creatinine, µmolL	70.43 ± 14.95	70.13 ± 17.50	0.9211	
Peak BNP, pg/mL	120.7 ± 160.6	179.4 ± 243.5	0.1338	
Baseline Troponin T, µg/L	1.741 ± 2.620	2.380 ± 3.138	0.2877	
Peak Troponin T, µg/L	3.683 ± 2.480	5.293 ± 2.537	< 0.001	
HbA1c,%	6.174 ± 1.154	6.339 ± 1.438	0.2619	
Fasting Glucose, mmol/L	6.153 ± 2.588	6.380 ± 2.292	0.2944	
**Infarct-related artery**				
LAD, n (%)	19 (41.30%)	46(55.42%)	0.1245	
RCA, n (%)	19 (41.30%)	27 (32.53%)	0.3190	
LCX, n (%)	8 (17.39%)	10 (12.05%)	0.4015	
Multivessel disease, n (%)	2 (4.35%)	2 (2.41%)	0.616	
Symptom-onset-to-balloon time (h)	6.698 ± 6.104	7.620 ± 8.533	0.1714	
FMC to wire time (h)	2.218 ± 1.532	2.759 ± 1.920	0.0744	
Initial TIMI flow grade > 1, n (%)	11 (23.91%)	15 (18.07%)	0.4283	
Final TIMI flow grade 3, n (%)	41 (89.13%)	73 (87.95%)	0.8415	
Syntax score	15.36 ± 9.215	16.72 ± 7.504	0.1223	
Stent implantation, n (%)	41 (89.13%)	78 (93.98%)	0.3242	
No. of stent per patients	1.130 ± 0.6535	1.241 ± 0.5757	0.2042	
Stent length (mm)	34.66 ± 17.06	34.63 ± 14.70	0.8133	
Stent diameter (mm)	3.097 ± 0.45	3.072 ± 0.46	0.5842	
Collateral circulation, n (%)	5 (10.87%)	9 (10.84%)	0.9963	

WBC, white blood cells; CRP, C-reactive protein; NLR, neutrophil lymphocyte ratio; LDL-C, ow-density lipoprotein cholesterol; HbA1c, glycosylated hemoglobin A1c; BNP, B-type natriuretic peptide; LCX, left circumflex artery; LAD, left anterior descending coronary artery; FMC, first medical contact; RCA, right coronary artery; TIMI, thrombolysis in myocardial infarction; Data are presented as mean ± SD or as number (%) affected

**Table 3:** CMR and adipose tissue characteristics between patients with or without MVO

Parameter	Patients without MVO, *n* = 46	Patients with MVO, *n* = 83	*P* value
**CMR parameters**			
LVEF, %	55.63 ± 6.941	43.31 ± 9.855	< 0.001
CO, L/min	3.646 ± 0.7673	3.485 ± 0.9036	0.3090
LVDd, cm	5.300 ± 0.4624	5.379 ± 0.4803	0.1818
LVEDVI, mL/ m^2^	55.42 ± 14.11	64.80 ± 15.15	< 0.001
LVESVI, mL/ m^2^	25.05 ± 9.021	37.19 ± 12.31	< 0.001
Infarct size, % of LV mass	11.05 ± 8.894	24.55 ± 10.28	< 0.001
MVO, % of LV mass	0 ± 0	3.007 ± 2.741	< 0.001
**EAT thickness (mm)**			
Right ventricular free wall	5.226 ± 0.6962	5.449 ± 0.7749	0.1072
**Horizontal long-axis plane**			
Left AV groove	10.24 ± 1.387	11.86 ± 1.471	< 0.001
Right AV groove	12.95 ± 1.499	13.17 ± 1.636	0.4626
Anterior IV groove	6.711 ± 1.118	6.813 ± 1.258	0.6488
**Basal short-axis plane**			
Superior IV groove	9.389 ± 1.468	9.787 ± 1.164	0.1160
Inferior IV groove	6.072 ± 0.6735	6.235 ± 1.234	0.1554
Mean grooved segments	9.037 ± 0.7171	9.219 ± 0.7148	0.1701
**EAT cross-sectional area** **(cm** ^**2**^ **)**			
Horizontal long-axis plane	11.05 ± 1.852	11.27 ± 2.151	0.5620
**Short-axis plane**			
Basal	8.284 ± 2.057	9.878 ± 2.151	< 0.001
Mid-cavity	6.790 ± 1.612	7.373 ± 1.644	0.0545
Apical	4.470 ± 1.418	4.281 ± 1.411	0.3732
Mean	6.515 ± 1.408	7.177 ± 1.392	0.0111
LV EAT volume (mL)	37.20 ± 7.704	48.53 ± 9.920	< 0.001
EAT volume (mL)	55.10 ± 10.73	69.95 ± 15.42	< 0.001
LV EAT mass index (g/ m^2^)	18.63 ± 3.670	24.72 ± 5.049	< 0.001
EAT mass index (g/ m^2^)	27.59 ± 5.017	35.61 ± 7.703	< 0.001

CMR, cardiac magnetic resonance; LVEF, left ventricular ejection fraction; CO cardiac output; LVDd, left ventricular end diastolic dimension; LVEDVI, left ventricular end-diastolic volume index; LVESVI, left ventricular end-systolic volume index; LV, left ventricular; MVO, microvascular obstruction; AV, atrio-ventricular; IV, inter-ventricular; EAT, epicardial adipose tissue; LV EAT, left atrioventricular epicardial fat; Data are presented as mean ± SD

### Associations of EAT with clinical variables and MVO formation

**Fig. 2:**
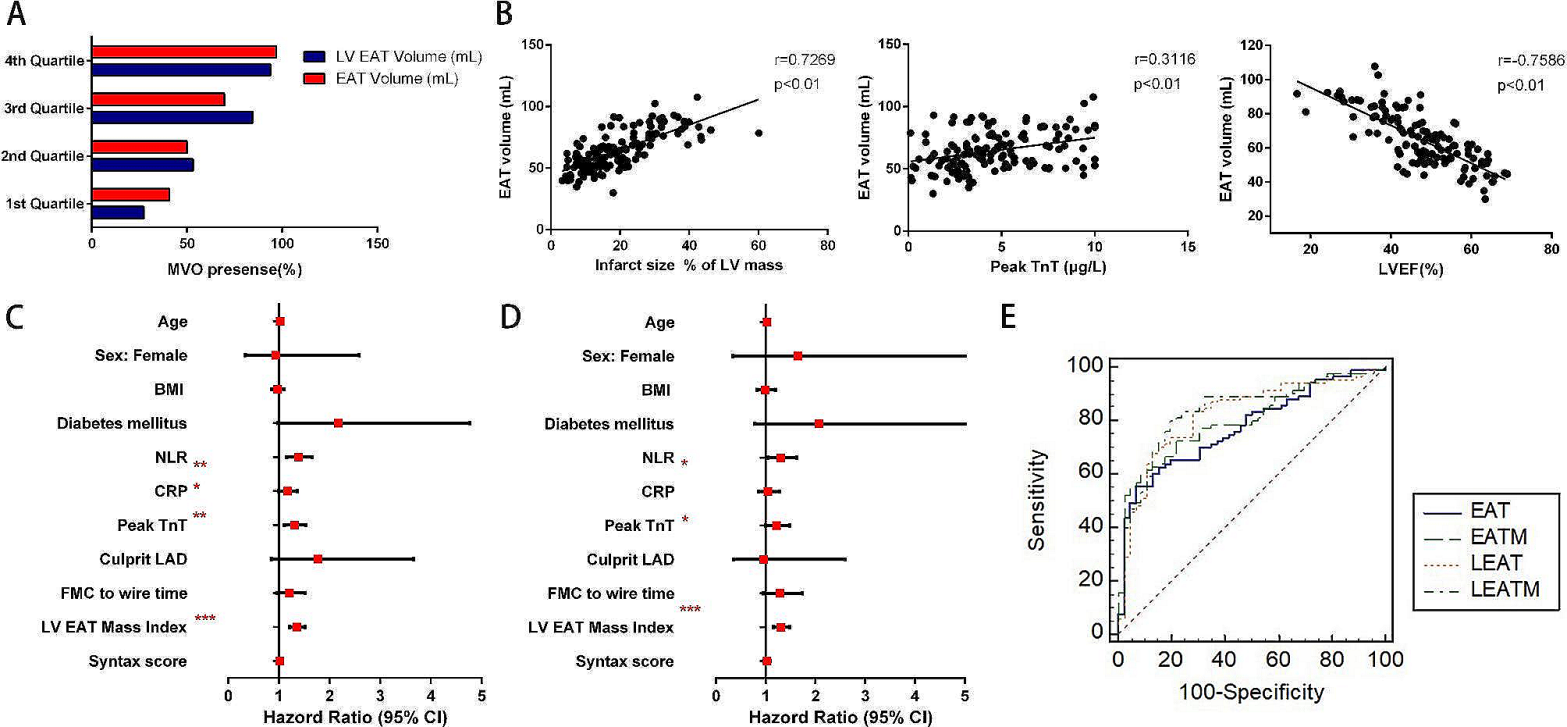
High EAT load was associated with MVO occurrence in STEMI patients. **A** Range distribution of MVO occurrence according to EAT and LV EAT volume. Bars represent the percentage of MVO occurrence identified at every quartile. **B** The association of EAT volume with infarct size, peak TnT and LVEF. **C** Univariate logistic regression analysis to identify the risk factors associated with the occurrence of MVO in patients with STEMI. **D** Multivariate logistic regression analysis to identify the risk factors associated with the occurrence of MVO in patients with STEMI. **E** Predictive accuracy of MVO based on ROC curve analysis. *P < 0.05, **P < 0.01, ***P < 0.001

Considering the asymmetric distribution of EAT, we measured the EAT thickness and cross-sectional area in different representative area according to previous study [37]. Significant increase of left atrioventricular (AV) groove fat thickness (11.86 ± 1.471 vs. 10.24 ± 1.387, *p* < 0.001) was observed in patients with MVO, whereas no such differences were found in other regional EAT thickness measurements. Moreover, In comparison to non-MVO patients, the cross-sectional area of EAT at basal level (9.878 ± 2.151 vs. 8.284 ± 2.057, *p* < 0.001) and the average of EAT cross-sectional area in short-axis planes (7.177 ± 1.392 vs. 6.515 ± 1.408, *p* = 0.0111) were larger in MVO group. We further calculated the total EAT volume and LV EAT volume and found that both EAT volume (69.95 ± 15.42 vs. 55.10 ± 10.73, *p* < 0.001) and LV EAT volume (48.53 ± 9.920 vs. 37.20 ± 7.704, *p* < 0.001) were significantly larger in subjects with MVO compared to subjects without MVO (Table 3). When we stratified the patients into four groups according to EAT volume or LV EAT volume using quartile method, the incidence of MVO was highest in patients with largest EAT volume (4th Quartile group), much higher than that of patients with smallest EAT volume (1th Quartile group) (Fig. 2A). We found that EAT volume significantly correlated with infarct size, peak TnT, while negatively correlated with LVEF% in STEMI patients. Neither age nor the complexity of coronary arteries was correlated with EAT volume (Fig. 2B and Fig. S2). After indexing EAT by BSA to exclude the correlation between BMI and EAT, the differences of EAT mass index and LV EAT mass index between MVO and non-MVO group were still apparently significant (Table 3).

Regarding factors associated with MVO formation, univariable analysis showed that NLR, CRP, peak TnT and LV EAT mass index reached statistical significance. Taking into account of all variables that clustered patients with MVO with the p value less than 0.10 and correcting for baseline demographic characteristics, multivariable analysis demonstrated that NLR, peak TnT and LV EAT mass index were independent predictors of MVO (Fig. 2C, D; Table 4). Additionally, the ROC curves demonstrated a superior performance of LV EAT mass index vs. EAT volume in predicting MVO formation (AUC: 0.835 vs. 0.775, *P* = 0.0184, Fig. 2E). In summary, LV EAT mass index showed a robust and independent relationship with MVO formation and a cutoff value > 21.5192 g/m^2^ predicted the presence of MVO with a sensitivity and specificity of 79.5% and 80.4%, respectively.

**Table 4:** Univariable and multivariable predictors of presence of MVO

	Univariable analysis		Multivariable analysis	
	OR (95% CI)	*P* value	OR (95% CI)	*P* value
Age, years	1.026 (0.994–1.059)	0.116		
Female	0.942 (0.343–2.587)	0.907		
BMI, kg/m^2^	0.977 (0.861–1.108)	0.714		
Diabetes mellitus	2.170 (0.987–4.774)	0.054	2.069 (0.782–5.479)	0.143
NLR	1.384 (1.152–1.662)	0.001	1.299 (1.041–1.621)	0.021
CRP, mmol/L	1.171 (1.002–1.367)	0.047	1.042 (0.852–1.275)	0.686
Peak Troponin T, µg/L	1.306 (1.110–1.538)	0.001	1.217 (1.002–1.478)	0.047
Culprit LAD	1.767 (0.852–3.663)	0.126		
FMC to wire time (h)	1.208 (0.959–1.521)	0.108		
LV EAT mass index (g/m^2^)	1.353 (1.209–1.514)	0.001	1.299 (1.155–1.460)	0.001
Syntax score	1.021 (0.976–1.069)	0.364		

NLR, neutrophil lymphocyte ratio; LAD, left anterior descending coronary artery; FMC, first medical contact; LV, left ventricular; EAT, epicardial adipose tissue

### Relationship between EAT accumulation and DPP4, GLP-1 and GLP-2

**Fig. 3:**
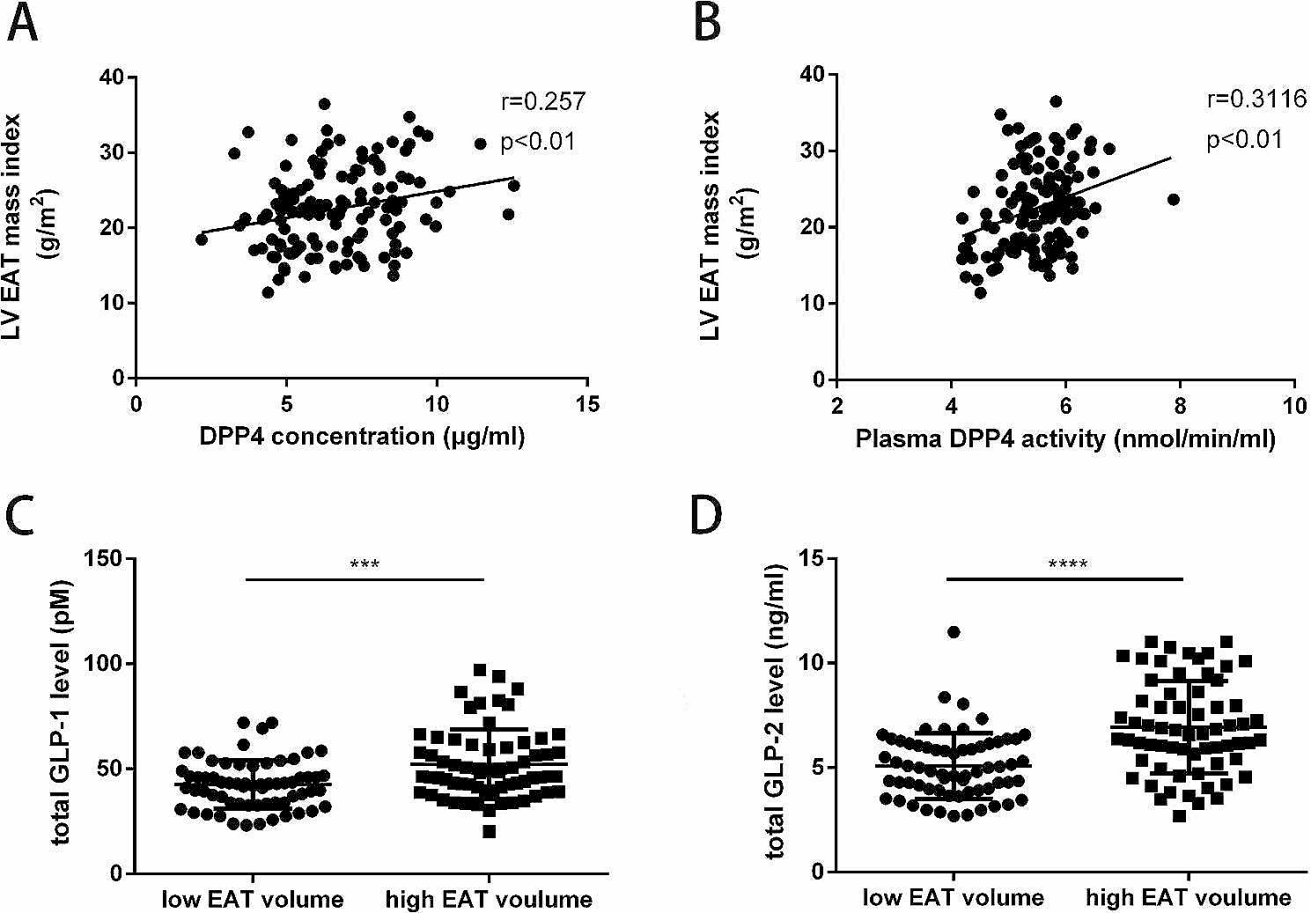
Association between EAT accumulation and DPP4, GLP-1 and GLP-2. **A** Correlation between LV EAT mass index and plasma DPP4 concentration in STEMI patients. **B** Correlation between LV EAT mass index and plasma DPP4 activity in STEMI patients. **C** Circulating level of total GLP-1 in STEMI patients. **D** Circulating level of total GLP-2 in STEMI patients. Data are presented as mean ± SD. ***P < 0.001, ****P < 0.0001

DPP4, a multifunctional type II membrane glycoprotein, plays an important role in maintaining metabolic balance and modulating inflammation. DPP4 also acts as a novel adipokine and is primarily secreted by the visceral adipose tissue into circulation, where it exerts autocrine and paracrine functions [38]. In our study, we measured plasma DPP4 concentration and DPP4 activity in STEMI patients and found that LV EAT mass index was positively correlated with circulating DPP4 and DPP4 activity (Fig. 3A, B). Significant but weak to moderate associations were found between plasma DPP4 and parameters associated with glucose metabolism, while no associations of plasma DPP4 with BMI and lipid metabolism parameters were found in the current study (Fig. S3).

The main enzymatic ability of DPP4 is to cleave N-terminal dipeptides from a variety of substrates including incretin hormones (GLP-1 and GLP-2) and make them inactive. Recent studies proposed that circulating levels of total GLP-1 and GLP-2 were increased in patients with AMI and were proved to be significant predictors of adverse cardiovascular prognosis [39–41]. Considering previous studies confirmed the expression of GLP-1 receptor (GLP-1R) and GLP-2 receptor (GLP-2R) in EAT [42, 43], EAT might be a potential target of GLP-1 and GLP-2. Our observation that higher levels of total GLP-1 and GLP-2 levels in those with larger volume of EAT might indicate the hypothesis of an underlying compensatory mechanism that try to compensate the reduction of the active form and modulate the EAT metabolism, which deserves further evaluation in large scale study (Fig. 3C, D).

### EAT depletion preserved cardiac function, attenuated MVO through alleviating cardiac inflammation following myocardial I/R injury

**Fig. 4:**
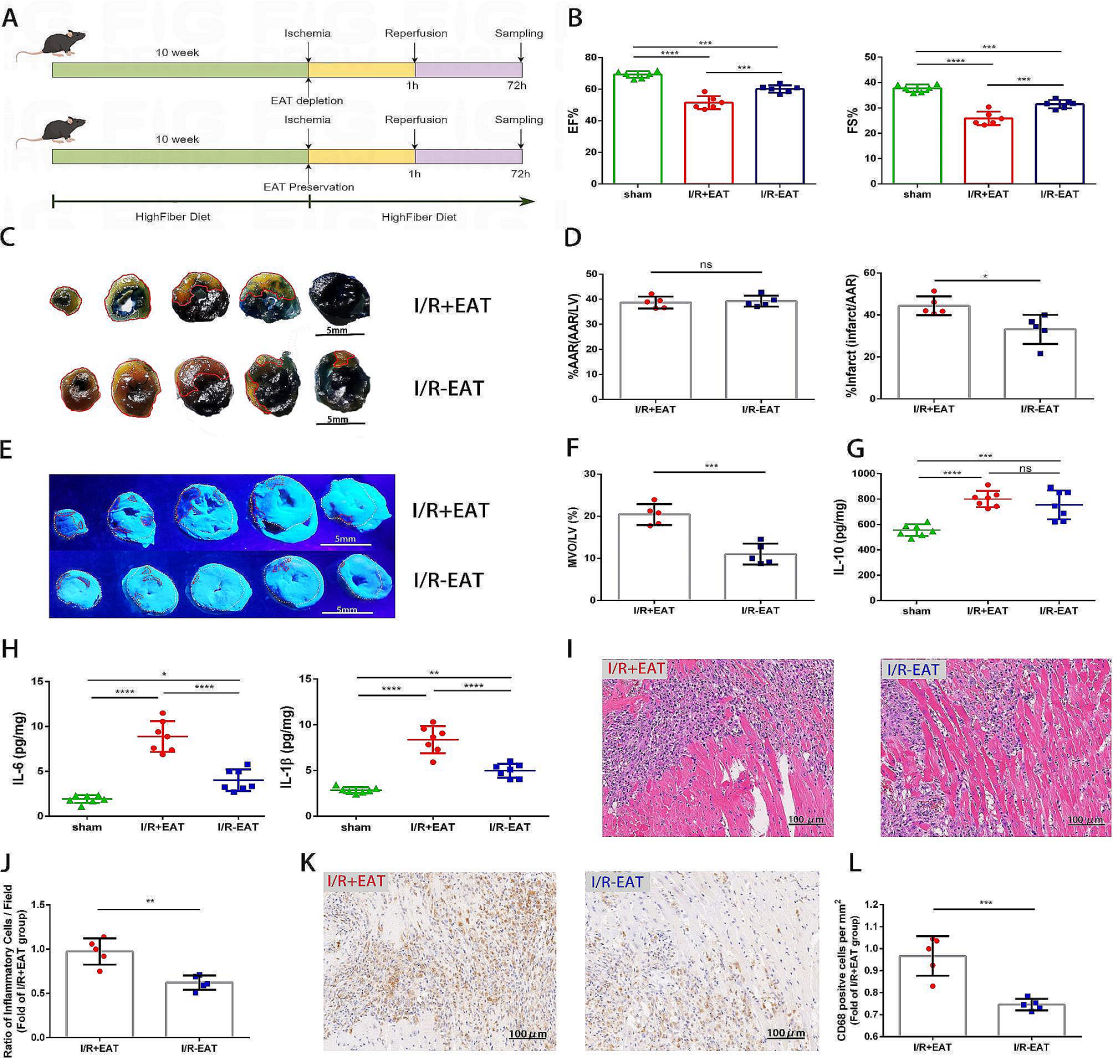
EAT depletion ameliorated microvascular dysfunction and cardiac inflammation in mice. **A** The experimental protocol for assessing the impact of excessive EAT accumulation on myocardial I/R injured mice. **B** Quantitative assessment of EF% and FS% using echocardiography 3 days following operation (*n* = 6). **C** Representative images of heart slices stained with Evans Blue and TTC from mice 3 days after myocardial I/R induction. The red line represents the area at risk (AAR) and the white dotted line represents the size of the infarct (IS). Scale bar = 5 mm. **D** Quantitative analysis of the percentage of AAR and percentage of infarct size in hearts in C (*n* = 5). **E** Representative thioflavin-S stained hearts isolated from myocardial I/R injured mice 1 day post operation with or without EAT. White dotted line represents the left ventricular area (LV) and red dotted line represents the size of MVO. Scale bar = 5 mm. **F** quantification of MVO percentage in hearts in E (*n* = 5). **G** Cytokine expression of IL-10 in the hearts of mice from different groups 3 days post I/R (*n* = 7). **H** Cytokine expression of IL-6 and IL-1β in the hearts of mice from different groups 3 days post I/R (*n* = 7). **I** Representative HE staining of I/R injured hearts from EAT-depletion and EAT preservation mice 3 days following operation. Scale bar = 100 μm. **J** Quantification of inflammatory cell infiltration (%) within the ischemic hearts in I (*n* = 5). **K** Immunohistochemical staining of CD68^+^ cells within the ischemic zone 3 days following I/R. Scale bar = 100 μm. **L** Quantification of CD68^+^ cells from K (*n* = 5). Data are presented as mean ± SD *P < 0.05, **P < 0.01, ***P < 0.001, ****P < 0.0001, ns = not significant

Given that high EAT volume was identified as an independent risk factor for MVO formation in STEMI patients in our observational study, we then explored the impact of EAT on MVO formation in a mouse model of myocardial I/R. To simulate the high EAT load status, high fat diet-fed C57BL/6 mice undergoing myocardial I/R injury were used to investigate the role of EAT depletion in myocardial I/R injury (Fig. 4A). A better preservation of cardiac function and a reduction in infarct size (IS/AAR) were found in EAT depletion mice three days post operation (Fig. 4B-D). Furthermore, thioflavin-S staining demonstrated that MVO size was apparently smaller in I/R-EAT group as compared with I/R + EAT group (Fig. 4E, F). All of these findings declared a direct causal relationship between EAT accumulation and the severity of myocardial I/R injury, especially MVO formation.

Due to the well-established immunomodulatory property of EAT [1, 8], we measured the levels of inflammatory cytokines in heart tissues and demonstrated that the concentration of IL-10 was comparable between two groups (Fig. 4G). The levels of IL-1β and IL‐6 in the heart tissues of EAT depletion mice were obviously lower versus the EAT preservation mice (Fig. 4H). Since these cytokines were mainly produced by immune cells, we performed Hematoxylin and eosin staining in hearts harvested three days following myocardial I/R and found reduced inflammatory cell infiltration in EAT depletion mice (Fig. 4I, J). To clarify the type of infiltrated inflammatory cells, we further used immunohistochemistry to detect the infiltration of immune cells within the ischemic region of hearts isolated three days following I/R. The quantity of CD68^+^ cells (marker of macrophages) was significantly reduced in the hearts of EAT depletion mice (Fig. 4K, L), which indicated that macrophages might be involved in the pathophysiological effects of EAT in MVO formation.

### Secretory products from EAT promoted macrophage M1 polarization under myocardial I/R injury

**Fig. 5:**
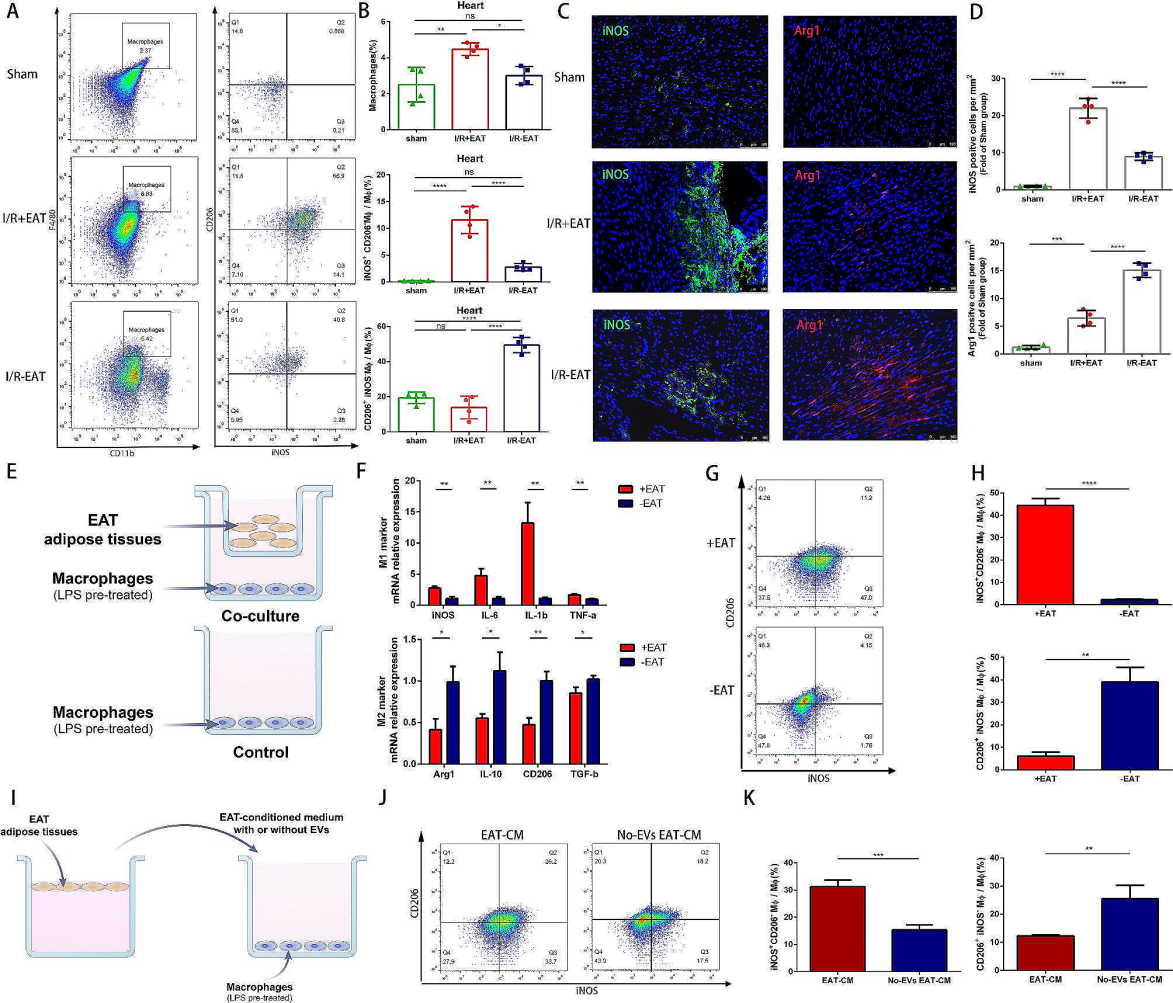
Excessive EAT accumulation promoted macrophage M1 polarization under myocardial I/R condition through secreting inflammatory EVs. **A** Representative flow cytometry plots depicting the ratio of total macrophage (CD11b^+^F4/80^+^), M1 phenotype (CD11b^+^F4/80^+^iNOS^+^CD206^−^) and M2 phenotype (CD11b^+^F4/80^+^iNOS^−^CD206^+^) in cardiac tissue of mice 3 days post-operation. **B** Quantification of flow cytometry data from A (*n* = 4). **C** Immunofluorescence staining of iNOS^+^ and Arg1^+^ cells within the ischemic zone respectively in mice 3 days post operation. **D** Quantification of iNOS^+^ and Arg1^+^ cells from groups defined in C (*n* = 4). **E** Experimental scheme of EAT and macrophages co-culture. **F** Gene expression patterns of M1 markers (iNOS, IL-6, TNFα and IL-1β) and M2 markers (Arg1, CD206, IL-10 and TGFβ) were analyzed in LPS-stimulated macrophages after co-culturing with EAT or not for 48 h (*n* = 3). **G** Representative flow cytometry plots indicating the percentages of M1 (iNOS^+^CD206^−^) and M2 (iNOS^−^CD206^+^) phenotype in macrophages. **H** Quantification of flow cytometry data in G (*n* = 3). **I** Experimental scheme of macrophages co-culturing with EAT-conditioned medium with or without EVs. **J** Representative flow cytometry plots indicating the percentages of M1 (iNOS^+^CD206^−^) and M2 (iNOS^−^CD206^+^) phenotype in different groups described above. **K** Quantification of flow cytometry data in J (*n* = 3). Data are presented as mean ± SD *P < 0.05, **P < 0.01, ***P < 0.001, ****P < 0.0001, ns = not significant

To explore the potential effect of EAT on macrophage, we collected the heart tissues surrounding infarct area 3 days after myocardial I/R injury. The quantification of macrophages and their classical (M1) or alternatively activated (M2) phenotypes were measured using flow cytometry. As expected, we observed that removal of EAT significantly reduced the total macrophage population, which is correspondent with CD68 staining results. In comparison to I/R + EAT mice, the proportion of inflammatory M1 macrophages was notably reduced, while the proportion of reparative M2 macrophages was significantly elevated in the I/R-EAT group, indicating that EAT played a pivotal role in maintaining the balance of M1 and M2 macrophages (Fig. 5A, B). To validate the findings that high EAT load promoted the transforming of inflammatory macrophages under myocardial I/R injury, we further examined the expression pattern of M1 and M2 macrophages associated markers in 3-day post I/R hearts. After EAT depletion, the qRT-PCR analysis revealed a significant decrease in M1 gene expression markers (iNOS, IL-6, TNF-α, and IL-1β) and a relevant increase in M2 markers (Arg1, IL-10, CD206, and TGF-β) (Fig. S4). Immunofluorescence staining also confirmed that removal of EAT resulted in significant increase of M2 macrophages (Arg1 positive) and decrease of M1 macrophages (iNOS positive), which was consistent with qPCR analysis (Fig. 5C, D). Collectively, these data demonstrated that high epicardial fat load aggravated cardiac inflammation under myocardial I/R condition via promoting macrophage M1 polarization.

To further identify the direct effects of EAT on macrophages, we utilized an in vitro model using the murine RAW 264.7 macrophage cell line to avoid the interference of multiple cells in vivo model (Fig. 5E). To induce an inflammatory microenvironment, LPS was added prior to co-culturing with EAT. We found that co-culturing with EAT notably increased the IL-6, IL1-β, TNFα and iNOS production and decreased the expression of IL-10, TGFβ, CD206 and Arg1 (Fig. 5F). Moreover, flow cytometry analysis clearly demonstrated that co-culturing with EAT increased the ratio of M1 maocrphages (iNOS^+^CD206^−^) and decreased the ratio of M2 macrophages (iNOS^−^CD206^+^) under LPS stimulation (Fig. 5G, H). To clarify which kind of secretory product contribute to the immunomodulatory effect of EAT on macrophages, macrophages were cultured in EAT-conditioned medium with extracellular vesicles (EVs) or without EVs (non-EV EAT-CM) (Fig. 5I). Similarly, exposure to EAT-CM significantly facilitated the polarization of macrophages towards inflammatory phenotype compared with macrophages treated with non-EV EAT-CM (Fig. 5J, K). These collective results supported the hypothesis that EAT-derived EVs played a potentially more critical role than soluble factors in macrophage polarization modulation.

### The cardioprotective effect of GLP-1 receptor agonist and GLP-1/GLP-2 receptor dual agonist might be partially achieved by targeting epicardial fat metabolism

**Fig. 6:**
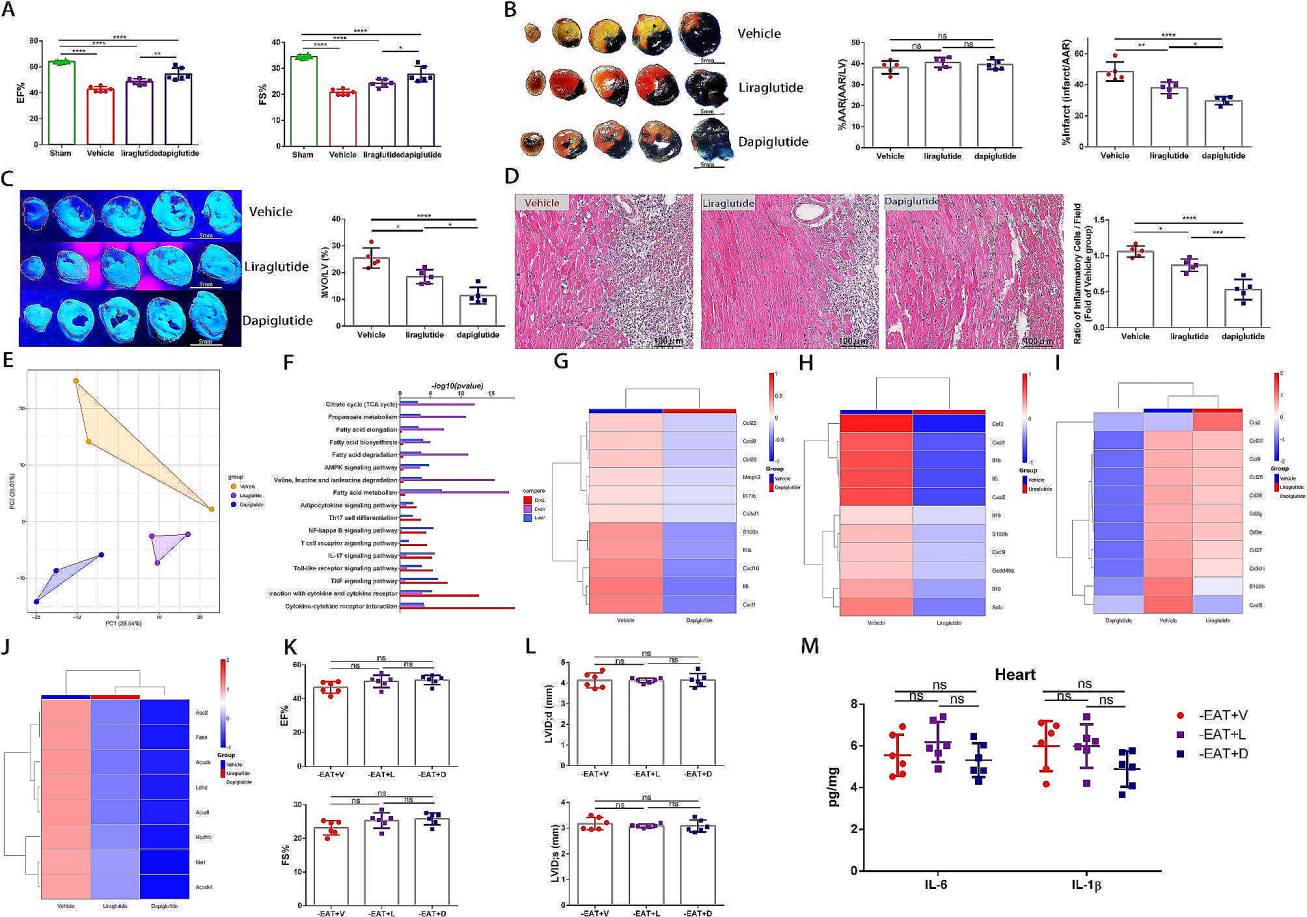
GLP-1 receptor agonist and GLP-1/GLP-2 receptor dual agonist attenuated myocardial I/R injury and alleviated cardiac inflammation through modulating EAT inflammatory and metabolic status. **A** Quantitative assessment of EF% and FS% in mice treated with vehicle, liraglutide and dapiglutide 3 days post-operation (*n* = 6). **B** Representative images of heart slices stained with Evans Blue&TTC and quantification of the percentage AAR and percentage infarct 3 days following I/R (*n* = 5). Scale bar = 5 mm. **C** Representative thioflavin-S stained heart slices and quantification of MVO percentage in vehicle, liraglutide and dapiglutide treated mice 1 day post operation (*n* = 5). Scale bar = 5 mm. **D** Representative images of H.E.stained hearts and quantification of inflammatory cell infiltration (%) 3 days after surgery (*n* = 5). Scale bar = 100 μm. **E** Principal components analysis of transcriptional profiling of EAT from vehicle, liraglutide and dapiglutide treated mice 3 days post I/R (*n* = 3). **F** Bar plots displaying the top significant KEGG pathways associated with DEGs in dapiglutide versus liraglutide, dapiglutide versus vehicle, and liraglutide versus vehicle groups. **G** Heatmap displaying expression patterns of top inflammation related DEGs between dapiglutide and vehicle group. **H** Heatmap displaying expression patterns of top inflammation related DEGs between liraglutide and vehicle group. **I** A heatmap depicting the expression patterns of top inflammation related DEGs among three groups. **J** A heatmap depicting the expression patterns of top metabolism related DEGs among three groups. **K** EF% and FS% of vehicle, liraglutide and dapiglutide treated I/R injured mice with EAT depletion 3 days following surgery (*n* = 6). **L** Quantitative assessment of LVIDd and LVIDs measured by echocardiography of vehicle, liraglutide and dapiglutide treated I/R injured mice with EAT depletion 3 days following I/R (*n* = 6). **M** Cardiac IL-6 and IL-1β levels of vehicle, liraglutide and dapiglutide treated I/R injured mice with EAT depletion 3 days after operation (*n* = 6). Data are presented as mean ± SD *P < 0.05, **P < 0.01, ***P < 0.001, ****P < 0.0001, ns = not significant

Recently, emerging evidence disclosed that the GLP-1R agonists liraglutide reduced EAT thickness to a greater extent than overall weight loss in individuals with type 2 diabetes and obesity [44, 45], suggesting a promising mechanism for the cardiovascular benefits of GLP-1 mimetics via EAT modulation. Our previous study further confirmed the therapeutic effects of GLP-2 analogue in myocardial I/R injury [17]. Considering the confirmation of both GLP-1R and GLP-2R expression in human EAT [42, 43], we then investigated the impact of GLP-1R agonists (liraglutide) and GLP-1/GLP-2 receptor dual agonist (dapiglutide) on myocardial I/R injury and how that influenced EAT metabolism. Both liraglutide and dapiglutide induced a significant reduction in EAT weight, mesenteric adipose tissues (MAT) weight and body weight relative to the vehicle group (Figure S6). Compared to vehicle group, liraglutide and dapiglutide efficiently preserved cardiac function, reduced infarct size and attenuated MVO under myocardial I/R injury (Fig. 6A-C). Additionally, significant reductions of inflammatory cells infiltration within ischemic heart were observed in liraglutide and dapiglutide treated mice (Fig. 6D). Dapiglutide group had smaller infarct size, higher EF% level and reduced inflammatory cells infiltration compared to liraglutide group, suggesting that GLP-1/GLP-2 receptor dual agonist might play a novel role on attenuation of myocardial I/R injury that is unlikely with single GLP-1R agonists.

We then investigated the gene expression patterns changes following liraglutide and dapiglutide treatment using bulk RNA sequence. According to our results, the transcriptome profiles significantly differed among the three groups (Fig. 6E). Inflammatory and metabolic genes were differentially regulated in both liraglutide and dapiglutide group compared with vehicle group (Fig. S5). According to KEGG analysis, most of the DEGs belonged to immune and lipid metabolism pathways, such as cytokine-cytokine receptor interaction, fatty acid biosynthesis, fatty acid degradation, amino acid metabolism, fatty acid metabolism and the AMPK signaling pathway (Fig. 6F). Liraglutide and dapiglutide treatment all induced a downregulation of genes involved in activation of myeloid and lymphoid cells like S100b, CXCL9, IL-6 and IL-1β relative to vehicle group, whereas the anti-inflammatory phenotype was more evident in dapiglutide group (Fig. 6G-I). Moreover, Dapiglutide induced a profound metabolic rewiring in EAT, with downregulation of genes involved in fatty acid metabolism (Acadl, Acadvl and Hadhb), fatty acid biosynthesis (Acacb and Fasn), adipogenesis (Me1) and glucose metabolism (Aco2, and Ldhd) (Fig. 6J).

To further verify whether the curative and immunomodulatory effect of liraglutide and dapiglutide were mediated by its impact on EAT, we performed EAT removal before treating the mice with liraglutide or dapiglutide as the above experimental design. Results demonstrated that the therapeutic effect of improving cardiac function and alleviating cardiac inflammation induced by liraglutide or dapiglutide disappeared after EAT depletion, suggesting that the protective effects of liraglutide and dapiglutide were at least partially ascribed to its impact on EAT modulation (Fig. 6K and L).

## Discussion

In our study, we used CMR to investigate the relationship of EAT distribution and quantity with MVO formation in patients with STEMI and firstly demonstrated that the increase of left atrioventricular EAT mass index was independently associated with MVO formation. We also demonstrated that increased circulating levels of DPP4 and high DPP4 activity seemed to be associated with EAT increase. Then we applied EAT depletion in mouse model of myocardial I/R and found that the promoting effect of high EAT load on MVO formation might largely rely on its modulation of cardiac inflammation. Our work further confirmed that EAT secretome aggravated cardiac inflammation under myocardial I/R condition via promoting macrophage M1 polarization. Furthermore, we demonstrated that the therapeutic effect of GLP-1 receptor agonist and GLP-1/GLP-2 receptor dual agonist in myocardial I/R injury were at least partially relied on its modulation of EAT.

Growing evidence declared that the increase of EAT volume was associated with coronary atherosclerosis progression and served as an independent predictor of future cardiovascular events in general population [3, 46, 47]. However, a few studies proposed the obesity paradox, suggesting that patients with higher volume of EAT presented better cardiac healing [10, 11]. These seemingly contradictory observations might be related to differences in race, inclusion criteria and measurements. Nevertheless, the association between EAT and the occurrence of MVO in STEMI patients has not been clearly clarified. In the current study, we first demonstrated that higher EAT mass index was associated with higher prevalence of MVO formation and a larger infarct size in individuals with STEMI. To elucidate the potential mechanism, we utilized an in vivo mouse model to investigate how EAT accumulation promoted MVO formation. In our work, we first found that infiltration of macrophages was reduced in the hearts of EAT depletion mice with smaller MVO size, suggesting that EAT-macrophages crosstalk might be involved in the pathological progress of MVO formation.

It is widely accepted that macrophage is a highly plastic and heterogeneous cell population, which comprises two typical subtype: inflammatory M1 macrophages and reparative M2 macrophages. The balance of pro-inflammatory M1 and anti-inflammatory M2 macrophages in I/R injured myocardium is critical for the duration and intensity of cardiac inflammation [34]. Thus, we further focused on the potential effects of EAT on macrophage phenotype transition and demonstrated that co-culture with EAT effectively shifted the polarization of macrophages towards pro-inflammatory state. There is no denying that the M1/M2 dichotomy is inadequate to describe the diverse phenotypes and various functions of macrophages in vivo [48]. However, this complexity and diversity of macrophages in vivo do not affect the conclusion that high EAT load acts as a pro-inflammatory mediator boosting the transition of macrophages towards inflammatory macrophages in myocardial I/R injury.

As an endocrine organ, EAT secretes different kinds of bioactive factors that mediate inter-organ crosstalk. Under normal physiologic conditions, EAT supports cardiovascular health by providing mechanical support, supplying energy and secreting a variety of adipokines. However, under pathologic circumstances, the excessive accumulation of EAT exacerbates cardiac injury by secreting pro-inflammatory adipokines through vasocrine or paracrine pathways [8, 49, 50]. In our study, by treating macrophages with EAT-CM with or without EVs, we demonstrated that the EAT modulated macrophages phenotype in an EV-dependent manner. EVs contain a variety of cargo components, such as miRNAs, lncRNA, proteins and lipids. Shaihov-Teper Olga and colleagues declared that EAT of patients with AF secreted a high amount of EVs that harbored a distinct proinflammatory, profbrotic, and proarrhythmic signature which accelerated the development of atrial myopathy and fibrillation in turn [8]. Besides, recent study demonstrated that human EAT derived miR-92a-3p prevented oxidative stress through targeting Wnt5a/Rac1/NADPH oxidase axis [49]. It was not yet clear whether miR-92a-3p was encapsulated into extracellular vesicles or worked independently. However, due to the extremely small volume of epicardial fat in rodent model [51], the isolation of sufficient EVs for animal experiments is hard to fulfill. Fully clarifying the decisive cargos in EAT derived EVs for macrophage modulation under myocardial I/R injury requires further study.

Considering the role of EAT accumulation in the pathological process of MVO formation, we screened for potential drugs for MVO prevention through targeting EAT induced cardiac inflammation. Recent clinical studies disclosed the same increasing tendency of total GLP-1 and GLP-2 in AMI patients, which were positively associated with inflammatory parameters, such as high-sensitivity CRP [39, 40]. Our cross-sectional cohort study further disclosed that both circulating DPP4 activity and the level of total GLP-1 and GLP-2 were higher in those with larger volume of EAT, suggesting a potential counter-regulatory mechanism that tried to compensate the reduction of active form and modulate the EAT metabolism. Although GLP-1 analogues resistant to DPP4 degradation are known to reduce the volume of EAT and exert cardioprotective effects [52], the effect and underlying mechanism of GLP-1 in MVO prevention remain elusive. Moreover, considering the confirmation of GLP-2 receptor expression in human EAT tissue [42], whether GLP-2 participates in the modulation of EAT metabolism remains unknown. Our findings first demonstrated that GLP-1R agonist and GLP-1/GLP-2 receptor dual agonist could prevent MVO formation under myocardial I/R injury through targeting ETA. We further revealed the synergistic effects of GLP-1/GLP-2 receptor dual agonist on EAT inflammation and metabolism, thus attenuating myocardial I/R injury. Our study demonstrated that GLP-1/GLP-2 receptor dual agonist targeting the interaction between EAT and cardiac inflammation might be a promising tool in preventing MVO formation and thus attenuating myocardial I/R injury.

### Study limitations

Our study had several limitations. Firstly, it was a single-center study that included limited number of patients. Secondly, this study was a cross-sectional study which made it challenging to derive causal associations between EAT and MVO formation. The results should be considered as exploratory and hypothesis-generating rather than conclusive. Follow-up studies may provide more conclusive findings. In the current study, we focused on the impact of EAT on macrophages modulation due to its critical role in cardiac inflammation. Further studies are needed to evaluate whether high EAT load has a direct effect on cardiomyoctes, endothelial cells or other immune cells. Furthermore, even though we have demonstrated that EAT-derived EVs might play a more critical role in macrophage polarization, the results of our study do not elucidate the precise modulatory mechanism that contributes to macrophage polarization. Future studies identifying the specific secretory products may be helpful in providing innovative and precise strategies for EAT targeting therapy to prevent MVO formation.

## Conclusion

Our work declares that excessive EAT accumulation contributes to the pathogenesis of MVO formation in STEMI patients and uncovers the involvement of EAT-derived EVs by which EAT promotes macrophage M1 polarization, thus exacerbating inflammatory injury. Moreover, our study sheds new light on the application of GLP-1/GLP-2 receptor dual agonist as a promising treatment targeting EAT modulation in myocardial I/R injury. Our findings highlight the EAT targeting therapy as a potential therapeutics strategy to prevent MVO formation and are of great translational value.

### Electronic supplementary material

Below is the link to the electronic supplementary material.

Supplementary Material 1

## Data Availability

No datasets were generated or analysed during the current study.

## Abbreviations

EAT: Epicardial adipose tissue
MVO: Microvascular obstruction
STEMI: ST-segment elevation myocardial infarction
CMR: Cardiac magnetic resonance
I/R: Ischemia/reperfusion
EFV: Epicardial fat volume
AMI: Acute myocardial infarction
pPCI: Primary percutaneous coronary intervention
CT: Computed Tomography
MI: Myocardial infarction
BMI: Body mass index
BSA: Body surface area
TnT: Troponin T
BNP: Brain natriuretic peptide
CRP: C-reactive protein
GLP-1: Glucagon-like peptide 1
GLP-2: Glucagon-like peptide 2
DPP4: Dipeptidyl peptidase-4
AMC: 7-amino-4-methylcoumarin
FMC: First medical contact
TIMI: Thrombolysis In Myocardial Infarction
SE: Spin-echo
B-TFE: Balanced turbo field echo
TE: Time of echo
TR: Time of repetition
FA: Flip angle
LGE: Late gadolinium enhancement
CO: Cardiac output
LVEDVI: Left ventricular end-diastolic volume index
LVESVI: Left ventricular end-systolic volume index
LVEF%: Left ventricular ejection fraction
LV: Left ventricular
FWHM: Full-width at half-maximum
LV EAT: Total left atrioventricular epicardial fat
ARRIVE: Animal research: reporting of in vivo experiments
HFD: High-fat diet
LAD: Left anterior descending coronary artery
LVIDs: Left ventricular internal diameter end-systolic
LVIDd: Left ventricular internal diameter end-diastolic
IVSs: Interventricular septal end-systolic thickness
IVSd: Interventricular septal end-diastolic thickness
LVPWd: Left ventricular posterior wall end-diastolic thickness
LVPWs: Left ventricular posterior wall end-systolic thickness
FS%: Fractional shortening
EF%: Ejection fraction
IS: Infarct size
TTC: 2,3,5-triphenyl-2 H-tetrazolium chloride
AAR: Area at risk
KCl: Potassium chloride
LPS: Lipopolysaccharide
EAT-CM: Conditioned media from EAT
ELISA: Enzyme-linked immunosorbent assay
SD: Standard deviation
ROC: Receiver-operating characteristic
GSEA: Gene set enrichment analysis
DEGs: Differentially expressed genes
KEGG: Kyoto Encyclopedia of Genes and Genomes
NLR: Neutrophil lymphocyte ratio
EV: Extracellular vesicles
CAD: Coronary artery disease
ACE-I: Angiotensin-converting enzyme inhibitors
ARB: Angiotensin II receptor blockers
LDL-C: Low-density lipoprotein cholesterol
RCA: Right Coronary Artery
LCX: Left Circumflex Branch
AV: Atrioventricular
IV: Interventricular
MAT: Mesenteric adipose tissues

## References

[1] Iacobellis G (2022). Epicardial adipose tissue in contemporary cardiology. Nat Rev Cardiol.

[2] Villasante Fricke AC, Iacobellis G (2019). Epicardial adipose tissue: clinical biomarker of cardio-metabolic risk. Int J Mol Sci.

[3] Christensen RH, von Scholten BJ, Hansen CS, Jensen MT, Vilsbøll T, Rossing P, Jørgensen PG (2019). Epicardial adipose tissue predicts incident cardiovascular disease and mortality in patients with type 2 diabetes. Cardiovasc Diabetol.

[4] Li Y, Liu B, Li Y, Jing X, Deng S, Yan Y, She Q (2019). Epicardial fat tissue in patients with diabetes mellitus: a systematic review and meta-analysis. Cardiovasc Diabetol.

[5] Madonna R, Massaro M, Scoditti E, Pescetelli I, De Caterina R (2019). The epicardial adipose tissue and the coronary arteries: dangerous liaisons. Cardiovasc Res.

[6] Conceição G, Martins D, Miranda M, Leite-Moreira I, Vitorino AF, Falcão-Pires R (2020). Unraveling the role of epicardial adipose tissue in coronary artery disease: partners in crime?. Int J Mol Sci.

[7] Zobel EH, Christensen RH, Winther SA, Hasbak P, Hansen CS, von Scholten BJ, Holmvang L, Kjaer A, Rossing P, Hansen TW (2020). Relation of cardiac adipose tissue to coronary calcification and myocardial microvascular function in type 1 and type 2 diabetes. Cardiovasc Diabetol.

[8] Shaihov-Teper O, Ram E, Ballan N, Brzezinski RY, Naftali-Shani N, Masoud R, Ziv T, Lewis N, Schary Y, Levin-Kotler LP (2021). Extracellular vesicles from epicardial fat facilitate atrial fibrillation. Circulation.

[9] Fisser C, Colling S, Debl K, Hetzenecker A, Sterz U, Hamer OW, Fellner C, Maier LS, Buchner S, Arzt M (2021). The impact of epicardial adipose tissue in patients with acute myocardial infarction. Clin Res Cardiol.

[10] Gohbara M, Iwahashi N, Akiyama E, Maejima N, Tsukahara K, Hibi K, Kosuge M, Ebina T, Umemura S, Kimura K (2016). Association between epicardial adipose tissue volume and myocardial salvage in patients with a first ST-segment elevation myocardial infarction: an epicardial adipose tissue paradox. J Cardiol.

[11] Bière L, Behaghel V, Mateus V, Assunção A, Gräni C, Ouerghi K, Grall S, Willoteaux S, Prunier F, Kwong R (2017). Relation of quantity of subepicardial adipose tissue to Infarct size in patients with ST-elevation myocardial infarction. Am J Cardiol.

[12] Heusch G (2019). Coronary microvascular obstruction: the new frontier in cardioprotection. Basic Res Cardiol.

[13] McCartney PJ, Eteiba H, Maznyczka AM, McEntegart M, Greenwood JP, Muir DF, Chowdhary S, Gershlick AH, Appleby C, Cotton JM (2019). Effect of low-dose intracoronary alteplase during primary percutaneous coronary intervention on microvascular obstruction in patients with acute myocardial infarction: a Randomized Clinical Trial. JAMA.

[14] Broch K, Anstensrud AK, Woxholt S, Sharma K, Tøllefsen IM, Bendz B, Aakhus S, Ueland T, Amundsen BH, Damås JK (2021). Randomized Trial of Interleukin-6 receptor inhibition in patients with acute ST-segment elevation myocardial infarction. J Am Coll Cardiol.

[15] de Waha S, Patel MR, Granger CB, Ohman EM, Maehara A, Eitel I, Ben-Yehuda O, Jenkins P, Thiele H, Stone GW (2017). Relationship between microvascular obstruction and adverse events following primary percutaneous coronary intervention for ST-segment elevation myocardial infarction: an individual patient data pooled analysis from seven randomized trials. Eur Heart J.

[16] Ghobrial M, Bawamia B, Cartlidge T, Spyridopoulos I, Kunadian V, Zaman A, Egred M, McDiarmid A, Williams M, Farag M (2023). Microvascular obstruction in Acute myocardial infarction, a potential therapeutic target. J Clin Med.

[17] Zhao J, Zhang Q, Cheng W, Dai Q, Wei Z, Guo M, Chen F, Qiao S, Hu J, Wang J (2023). Heart-gut microbiota communication determines the severity of cardiac injury after myocardial ischaemia/reperfusion. Cardiovasc Res.

[18] Du Y, Ji Q, Cai L, Huang F, Lai Y, Liu Y, Yu J, Han B, Zhu E, Zhang J (2016). Association between omentin-1 expression in human epicardial adipose tissue and coronary atherosclerosis. Cardiovasc Diabetol.

[19] Wu L, Dalal R, Cao CD, Postoak JL, Yang G, Zhang Q, Wang Z, Lal H, Van Kaer L (2019). IL-10-producing B cells are enriched in murine pericardial adipose tissues and ameliorate the outcome of acute myocardial infarction. Proc Natl Acad Sci U S A.

[20] Ji Q, Zhang J, Du Y, Zhu E, Wang Z, Que B, Miao H, Shi S, Qin X, Zhao Y (2017). Human epicardial adipose tissue-derived and circulating secreted frizzled-related protein 4 (SFRP4) levels are increased in patients with coronary artery disease. Cardiovasc Diabetol.

[21] Gruzdeva O, Uchasova E, Dyleva Y, Borodkina D, Akbasheva O, Belik E, Karetnikova V, Brel N, Kokov A, Kashtalap V (2018). Relationships between epicardial adipose tissue thickness and adipo-fibrokine indicator profiles post-myocardial infarction. Cardiovasc Diabetol.

[22] Bekkers SC, Backes WH, Kim RJ, Snoep G, Gorgels AP, Passos VL, Waltenberger J, Crijns HJ, Schalla S (2009). Detection and characteristics of microvascular obstruction in reperfused acute myocardial infarction using an optimized protocol for contrast-enhanced cardiovascular magnetic resonance imaging. Eur Radiol.

[23] Ng ACT, Strudwick M, van der Geest RJ, Ng ACC, Gillinder L, Goo SY, Cowin G, Delgado V, Wang WYS, Bax JJ (2018). Impact of epicardial adipose tissue, left ventricular myocardial fat content, and interstitial fibrosis on myocardial contractile function. Circ Cardiovasc Imaging.

[24] Henningsson M, Brundin M, Scheffel T, Edin C, Viola F, Carlhäll CJ (2020). Quantification of epicardial fat using 3D cine Dixon MRI. BMC Med Imaging.

[25] Ibanez B, James S, Agewall S, Antunes MJ, Bucciarelli-Ducci C, Bueno H, Caforio ALP, Crea F, Goudevenos JA, Halvorsen S (2018). 2017 ESC guidelines for the management of acute myocardial infarction in patients presenting with ST-segment elevation: the Task Force for the management of acute myocardial infarction in patients presenting with ST-segment elevation of the European Society of Cardiology (ESC). Eur Heart J.

[26] Li JW, Chen YD, Chen WR, You Q, Li B, Zhou H, Zhang Y, Han TW (2017). Prognostic value of plasma DPP4 activity in ST-elevation myocardial infarction. Cardiovasc Diabetol.

[27] Sarkar J, Nargis T, Tantia O, Ghosh S, Chakrabarti P (2019). Increased plasma dipeptidyl Peptidase-4 (DPP4) activity is an obesity-independent parameter for glycemic deregulation in type 2 diabetes patients. Front Endocrinol (Lausanne).

[28] Kramer CM, Barkhausen J, Bucciarelli-Ducci C, Flamm SD, Kim RJ, Nagel E (2020). Standardized cardiovascular magnetic resonance imaging (CMR) protocols: 2020 update. J Cardiovasc Magn Reson.

[29] Wang TD, Lee WJ, Shih FY, Huang CH, Chen WJ, Lee YT, Shih TT, Chen MF (2010). Association of epicardial adipose tissue with coronary atherosclerosis is region-specific and independent of conventional risk factors and intra-abdominal adiposity. Atherosclerosis.

[30] Petrini M, Alì M, Cannaò PM, Zambelli D, Cozzi A, Codari M, Malavazos AE, Secchi F, Sardanelli F (2019). Epicardial adipose tissue volume in patients with coronary artery disease or non-ischaemic dilated cardiomyopathy: evaluation with cardiac magnetic resonance imaging. Clin Radiol..

[31] Reiner J, Thiery J, Held J, Berlin P, Skarbaliene J, Vollmar B, Jaster R, Eriksson PO, Lamprecht G, Witte M (2022). The dual GLP-1 and GLP-2 receptor agonist dapiglutide promotes barrier function in murine short bowel. Ann N Y Acad Sci.

[32] Kim ER, Park JS, Kim JH, Oh JY, Oh IJ, Choi DH, Lee YS, Park IS, Kim S, Lee DH (2022). A GLP-1/GLP-2 receptor dual agonist to treat NASH: targeting the gut-liver axis and microbiome. Hepatology.

[33] Bruen R, Curley S, Kajani S, Lynch G, O’Reilly ME, Dillon ET, Brennan EP, Barry M, Sheehan S, McGillicuddy FC (2019). Liraglutide attenuates preestablished atherosclerosis in apolipoprotein E-deficient mice via regulation of immune cell phenotypes and proinflammatory mediators. J Pharmacol Exp Ther.

[34] Zhao J, Li X, Hu J, Chen F, Qiao S, Sun X, Gao L, Xie J, Xu B (2019). Mesenchymal stromal cell-derived exosomes attenuate myocardial ischaemia-reperfusion injury through mir-182-regulated macrophage polarization. Cardiovasc Res.

[35] Wang J, Li L, Zhang Z, Zhang X, Zhu Y, Zhang C, Bi Y (2022). Extracellular vesicles mediate the communication of adipose tissue with brain and promote cognitive impairment associated with insulin resistance. Cell Metab.

[36] DeLong ER, DeLong DM, Clarke-Pearson DL (1988). Comparing the areas under two or more correlated receiver operating characteristic curves: a nonparametric approach. Biometrics.

[37] Wang TD, Lee WJ, Shih FY, Huang CH, Chang YC, Chen WJ, Lee YT, Chen MF (2009). Relations of epicardial adipose tissue measured by multidetector computed tomography to components of the metabolic syndrome are region-specific and independent of anthropometric indexes and intraabdominal visceral fat. J Clin Endocrinol Metab.

[38] Barchetta I, Cimini FA, Dule S, Cavallo MG (2022). Dipeptidyl Peptidase 4 (DPP4) as a Novel Adipokine: role in metabolism and Fat Homeostasis. Biomedicines.

[39] Kahles F, Rückbeil MV, Mertens RW, Foldenauer AC, Arrivas MC, Moellmann J, Lebherz C, Biener M, Giannitsis E, Katus HA (2020). Glucagon-like peptide 1 levels predict cardiovascular risk in patients with acute myocardial infarction. Eur Heart J.

[40] Kahles F, Mertens RW, Rueckbeil MV, Arrivas MC, Moellmann J, Lebherz C, Biener M, Giannitsis E, Katus HA, Marx N (2020). The gut hormone GLP-2 predicts cardiovascular risk in patients with acute myocardial infarction. Eur Heart J..

[41] Elbaz-Greener G, Bloch O, Kumets I, Blatt A, Rapoport MJ (2019). Endogenous glucagon-like peptide-1 system response is impaired during ST-elevation myocardial infarction in type 2 diabetes patients. Diabetes Obes Metab.

[42] Dozio E, Vianello E, Malavazos AE, Tacchini L, Schmitz G, Iacobellis G, Corsi Romanelli MM (2019). Epicardial adipose tissue GLP-1 receptor is associated with genes involved in fatty acid oxidation and white-to-brown fat differentiation: a target to modulate cardiovascular risk?. Int J Cardiol.

[43] Malavazos AE, Iacobellis G, Dozio E, Basilico S, Di Vincenzo A, Dubini C, Menicanti L, Vianello E, Meregalli C, Ruocco C (2023). Human epicardial adipose tissue expresses glucose-dependent insulinotropic polypeptide, glucagon, and glucagon-like peptide-1 receptors as potential targets of pleiotropic therapies. Eur J Prev Cardiol.

[44] van Eyk HJ, Paiman EHM, Bizino MB, de Heer P, Geelhoed-Duijvestijn PH, Kharagjitsingh AV, Smit JWA, Lamb HJ, Rensen PCN, Jazet IM (2019). A double-blind, placebo-controlled, randomised trial to assess the effect of liraglutide on ectopic fat accumulation in south Asian type 2 diabetes patients. Cardiovasc Diabetol.

[45] Myasoedova VA, Parisi V, Moschetta D, Valerio V, Conte M, Massaiu I, Bozzi M, Celeste F, Leosco D, Iaccarino G (2023). Efficacy of cardiometabolic drugs in reduction of epicardial adipose tissue: a systematic review and meta-analysis. Cardiovasc Diabetol.

[46] Cosson E, Nguyen MT, Rezgani I, Berkane N, Pinto S, Bihan H, Tatulashvili S, Taher M, Sal M, Soussan M (2021). Epicardial adipose tissue volume and myocardial ischemia in asymptomatic people living with diabetes: a cross-sectional study. Cardiovasc Diabetol.

[47] Brandt V, Decker J, Schoepf UJ, Varga-Szemes A, Emrich T, Aquino G, Bayer RR, Carson L, Sullivan A, Ellis L (2022). Additive value of epicardial adipose tissue quantification to coronary CT angiography-derived plaque characterization and CT fractional flow reserve for the prediction of lesion-specific ischemia. Eur Radiol.

[48] Martinez FO, Gordon S (2014). The M1 and M2 paradigm of macrophage activation: time for reassessment. F1000Prime Rep.

[49] Carena MC, Badi I, Polkinghorne M, Akoumianakis I, Psarros C, Wahome E, Kotanidis CP, Akawi N, Antonopoulos AS, Chauhan J (2023). Role of human epicardial adipose tissue-derived miR-92a-3p in myocardial redox state. J Am Coll Cardiol.

[50] Hao S, Sui X, Wang J, Zhang J, Pei Y, Guo L, Liang Z (2021). Secretory products from epicardial adipose tissue induce adverse myocardial remodeling after myocardial infarction by promoting reactive oxygen species accumulation. Cell Death Dis.

[51] Yamaguchi Y, Cavallero S, Patterson M, Shen H, Xu J, Kumar SR, Sucov HM (2015). Adipogenesis and epicardial adipose tissue: a novel fate of the epicardium induced by mesenchymal transformation and PPARγ activation. Proc Natl Acad Sci U S A.

[52] García-Vega D, Sánchez-López D, Rodríguez-Carnero G, Villar-Taibo R, Viñuela JE, Lestegás-Soto A, Seoane-Blanco A, Moure-González M, Bravo SB, Fernández ÁL (2024). Semaglutide modulates prothrombotic and atherosclerotic mechanisms, associated with epicardial fat, neutrophils and endothelial cells network. Cardiovasc Diabetol.

