# Supplementary material for: Excessive accumulation of epicardial adipose tissue promotes microvascular obstruction formation after myocardial ischemia/reperfusion through modulating macrophages polarization
Source: Cardiovasc Diabetol. 2024 Jul 5;23:236. doi: 10.1186/s12933-024-02342-8 (PMC11227217; doi:10.1186/s12933-024-02342-8)
Supplement: Supplementary file 1 — Supplementary Material 1 [file 12933_2024_2342_MOESM1_ESM.docx]

**Excessive Accumulation of Epicardial Adipose Tissue Promotes Microvascular Obstruction Formation after Myocardial Ischemia/Reperfusion through Modulating Macrophages Polarization**

**Jinxuan Zhao ^a #^; Wei Cheng ^b #^; Yang Dai ^c #^; Yao Li ^c^; Yuting Feng ^a^; Ying Tan ^a^; Qiucang Xue** ^d^**; Xue Bao ^a^; Xuan Sun ^a^; Lina Kang ^a^ ^*^; Dan Mu ^d *^; Biao Xu ^c *^**

^a^ Department of Cardiology, Nanjing Drum Tower Hospital, The Affiliated Hospital of Nanjing University Medical School, MOE Key Laboratory of Model Animal for Disease Study, Nanjing University, China;

^b^ Division of Colorectal Surgery, Department of General Surgery, Nanjing Drum Tower Hospital, The Affiliated Hospital of Nanjing University Medical School, Nanjing University, China;

^c^ Department of Cardiology, Nanjing Drum Tower Hospital, Clinical College of Nanjing Medical University, China;

^d^ Department of Radiology, Nanjing Drum Tower Hospital, The Affiliated Hospital of Nanjing University Medical School, Nanjing University, China;

# Jinxuan Zhao, Wei Cheng and Yang Dai contributed equally to this article.

*Corresponding Author: Biao Xu (xubiao62@nju.edu.cn), Dan Mu (mudan118@126.com), Lina Kang (kanglina@njglyy.com)

**SUPPLEMENTARY FIGURE LEGEND**


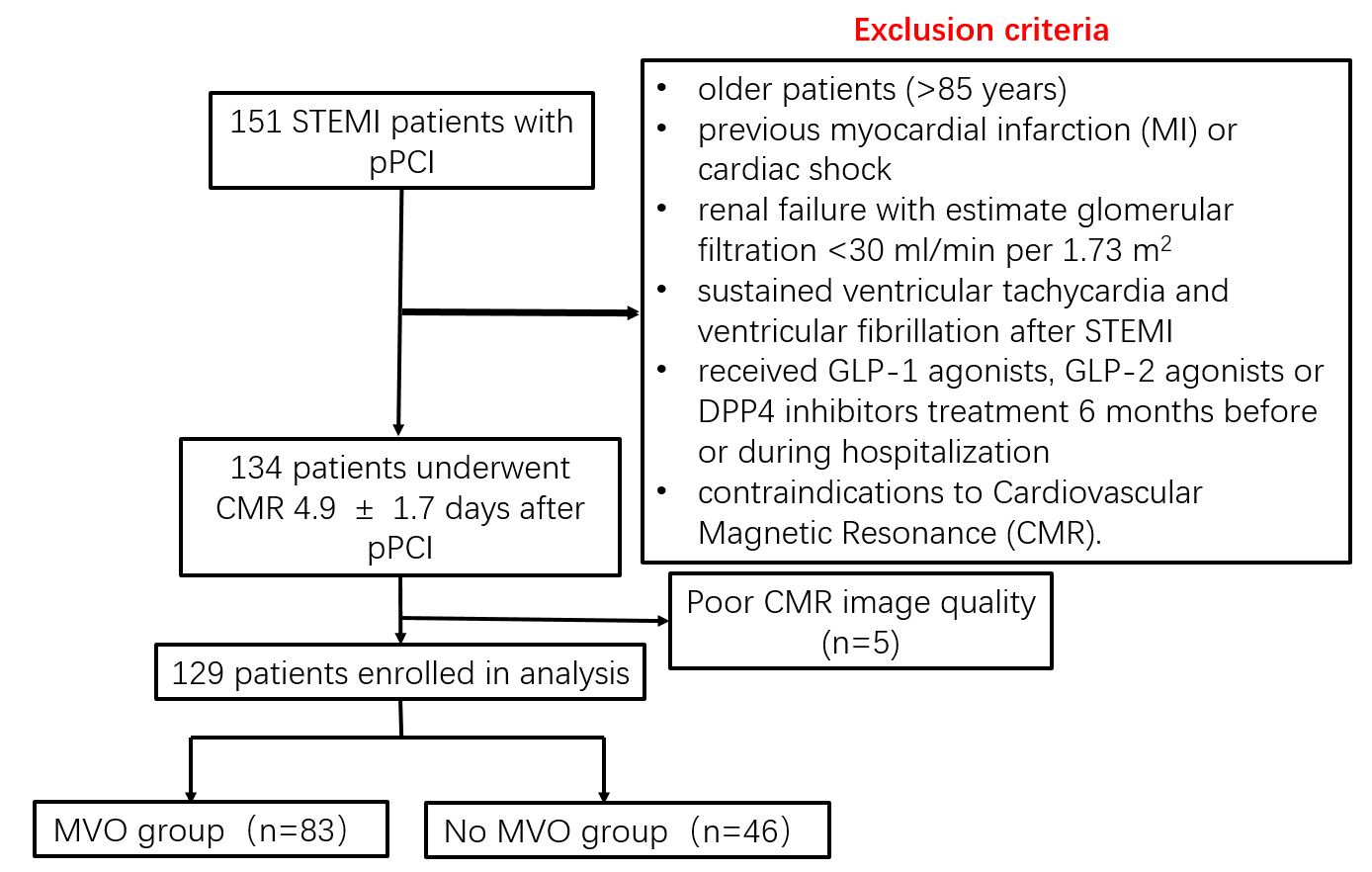


**Figure S1. Study flow chart. 129 patients were enrolled in the present study.**

**
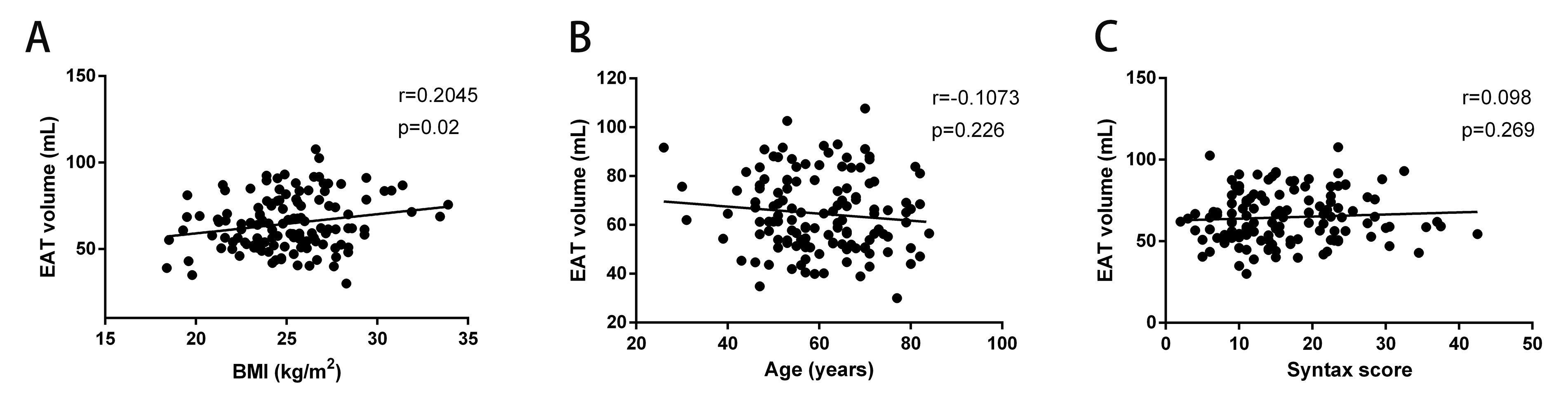
**

**Figure S2. The association of EAT volume with BMI, age and syntax score in STEMI patients.**

**
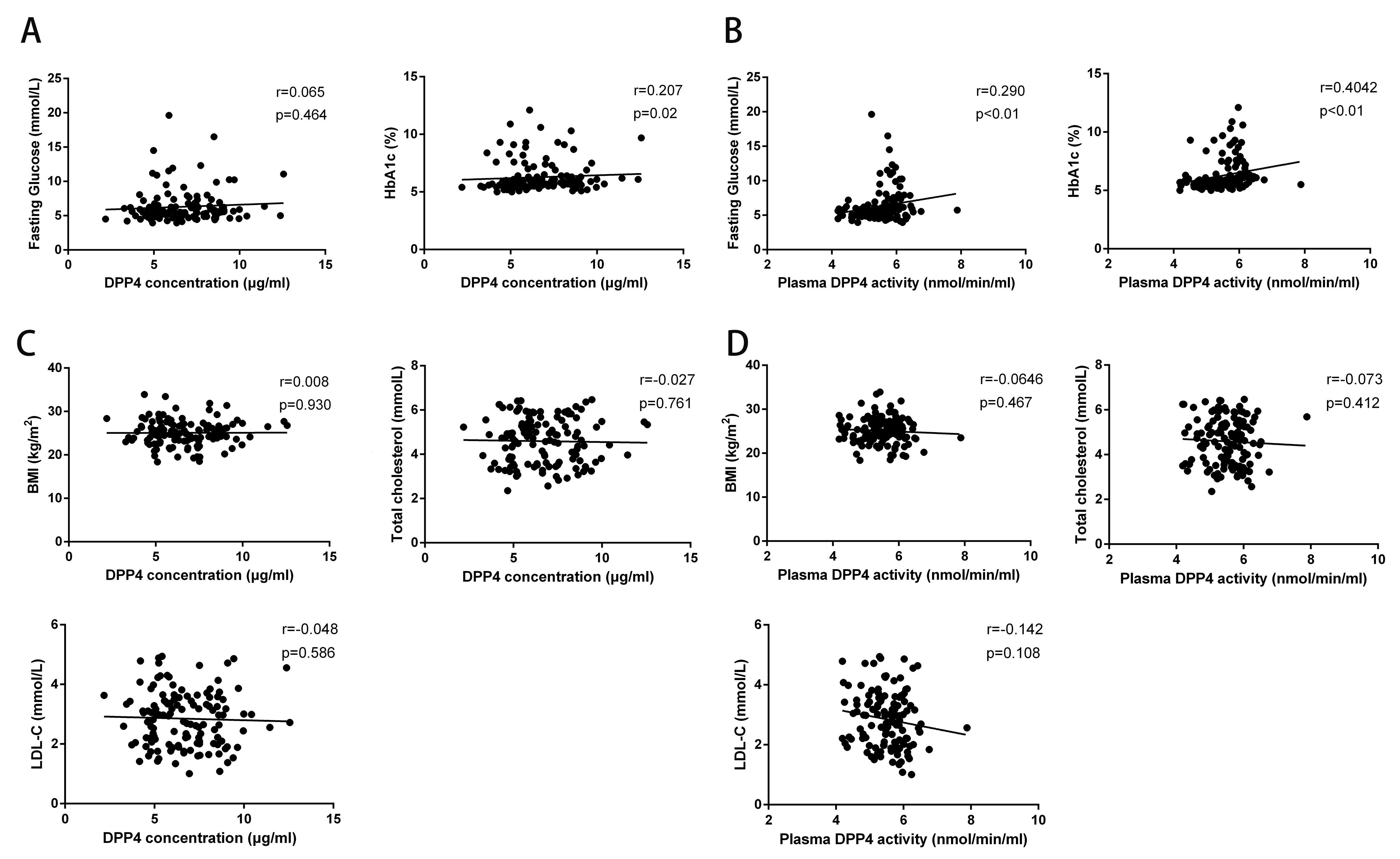
**

**Figure S3. The association of plasma DPP4 with glucose metabolism and lipid metabolism** **parameters in STEMI patients. A** Correlations between DPP4 concentration and parameters assessing glucose metabolism. **B** Correlations between DPP4 activity and parameters assessing glucose metabolism. **C** Correlations of DPP4 concentration with BMI and lipid metabolism parameters. **D** Correlations of DPP4 activity with BMI and lipid metabolism parameters. Correlation coefficients are reported as Spearman correlations.


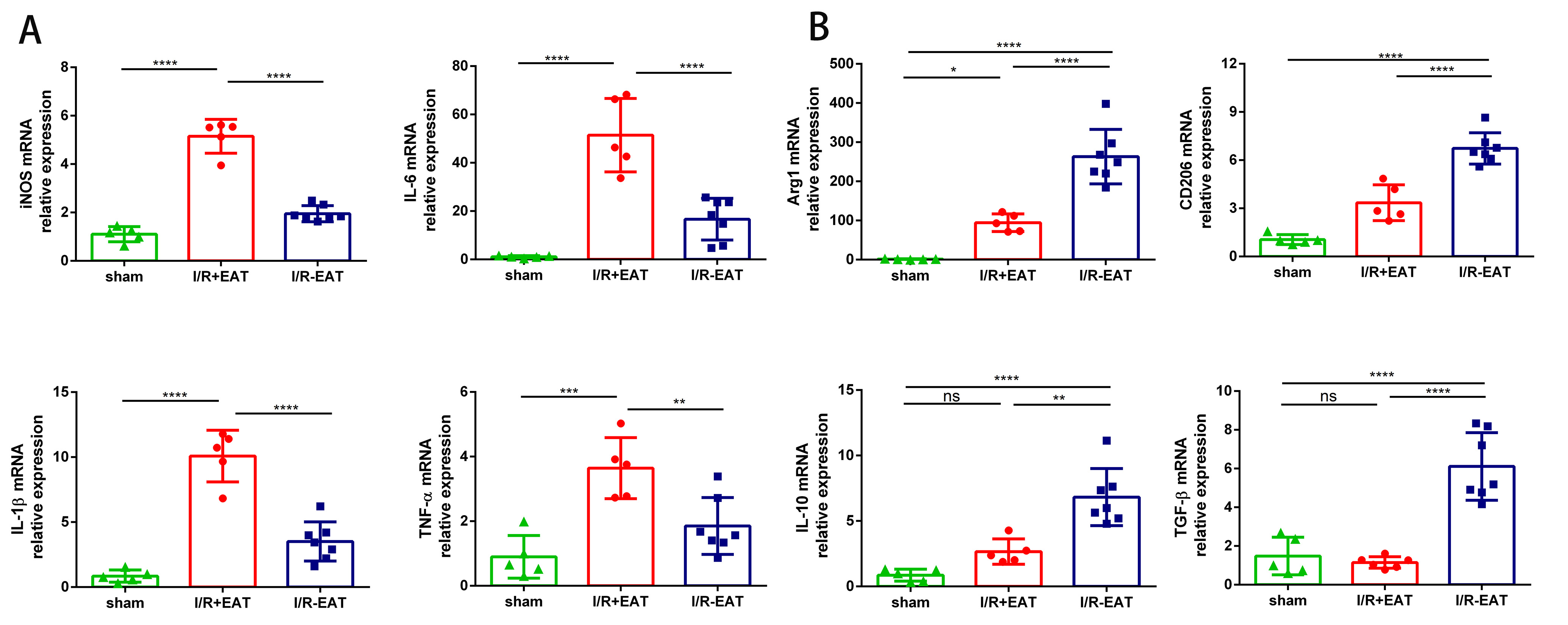


**Figure S4. Gene expression profiles of M1 markers and M2 markers in hearts of mice following myocardial I/R injury. A** Gene expression profiles of M1 markers (iNOS, IL-1β, IL-6, and TNF-α) in the hearts of mice sacrificed 3 days post operation (n=4-7). **B** Gene expression profiles of M2 markers (Arg1, IL-10, CD206 and TGF-β) in the hearts of mice sacrificed 3 days post operation (n=4-7). Graphs depict mean ± SD. *P < 0.05, **P < 0.01. ***P < 0.001, ****P < 0.0001, ns, not significant.


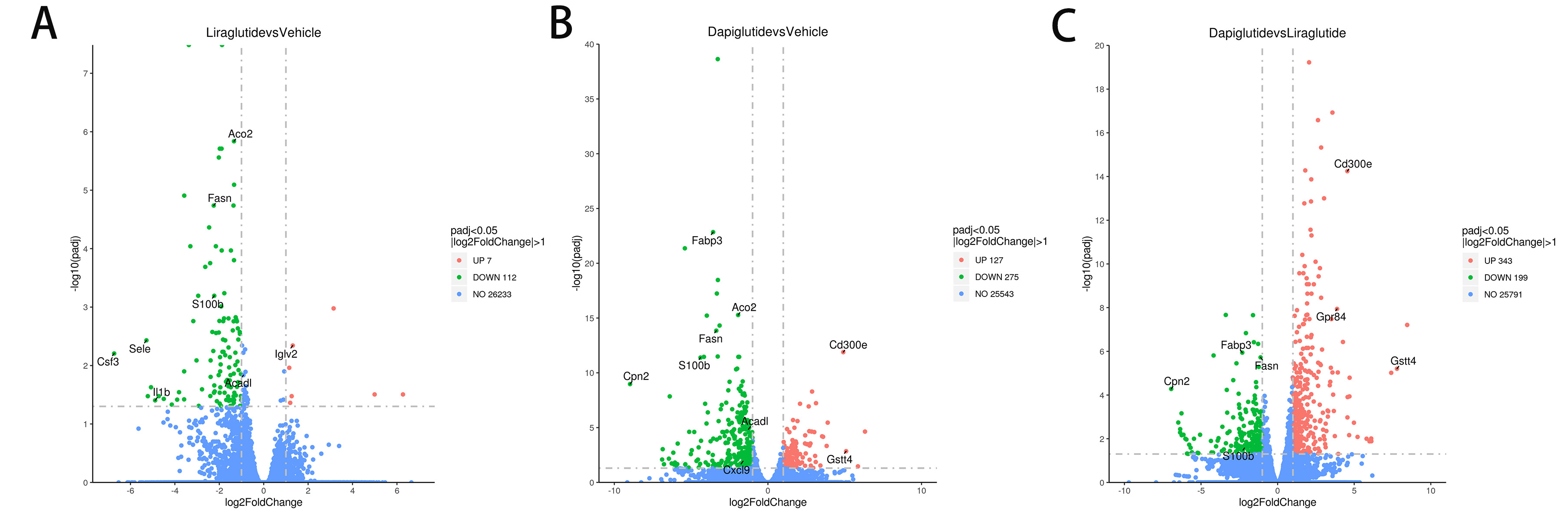


**Figure S5. Volcano plots of differentially expressed genes among Vehicle, Liraglutide and Dapiglutide group. A** Volcano plots illustrating the DEGs of EAT between Liraglutide and Vehicle group. **B** Volcano plots illustrating the DEGs of EAT between Dapiglutide and Vehicle group. **C** Volcano plots illustrating the DEGs of EAT between Dapiglutide and Liraglutide group.


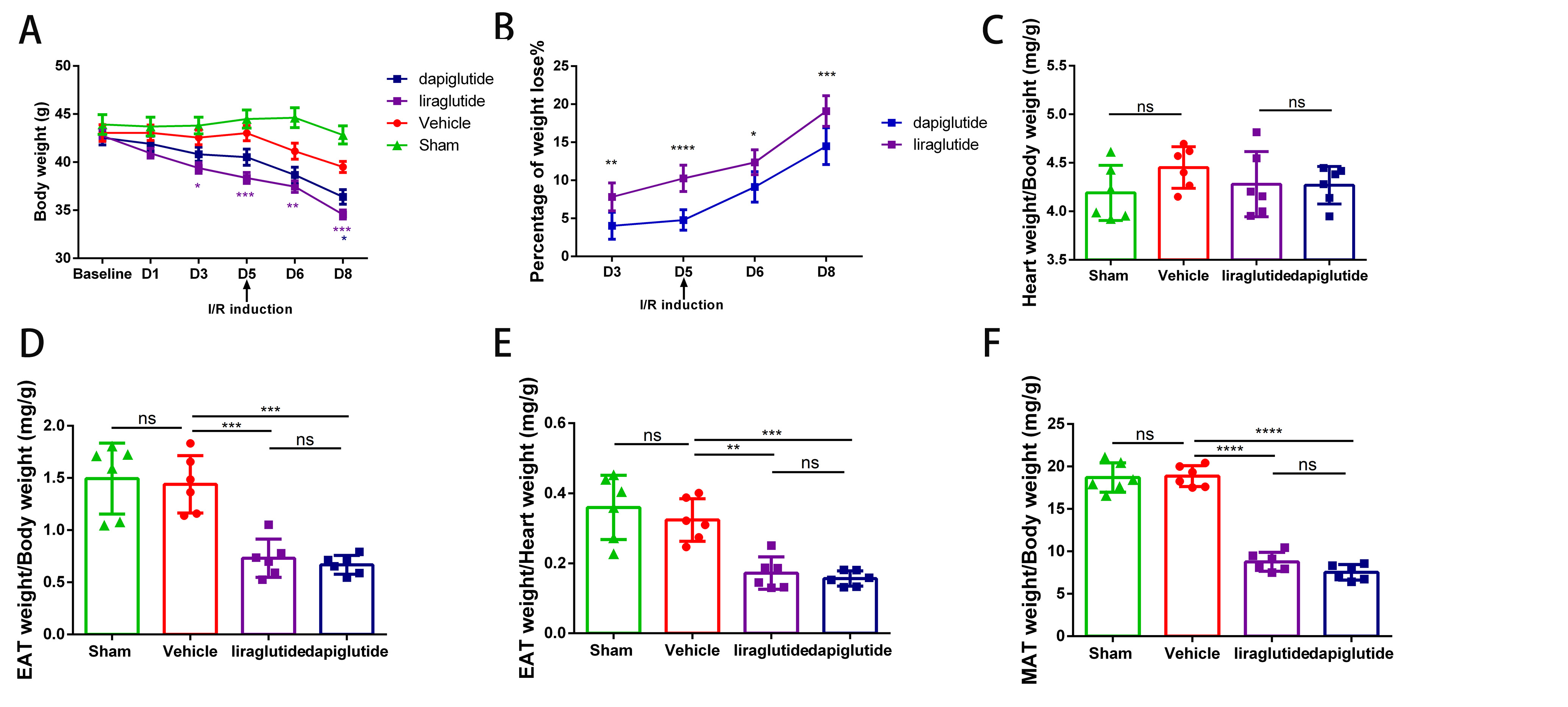


**Figure S6. Liraglutide and Dapiglutide reduced body weight and fat accumulation in myocardial I/R injured mice. A** Body weight changes following liraglutide and dapiglutide treatment (n=6). **B** Percentage of weight loss following liraglutide and dapiglutide treatment (n=6). **C** heart weight to body weight ratio among groups 3 days after surgery (n=6). **D** EAT weight to body weight ratio among groups 3 days post I/R (n=6). **E** EAT weight to heart weight ratio among groups 3 days post I/R (n=6). **F** Mesenteric adipose tissues (MAT) weight to body weight ratio among groups 3 days following I/R (n=6). Graphs depict mean ± SD. *P < 0.05, **P < 0.01. ***P < 0.001, ****P < 0.0001, ns, not significant.

SUPPLEMENTAL TABLES

**Supplemental Table 1 List of primers used for quantitative real‐time PCR**

| Gene | Forward primer | Reverse primer |
| --- | --- | --- |
| Mouse TNFα | AACTCCAGGCGGTGCCTATG | TCCAGCTGCTCCTCCACTTG |
| Mouse IL-10 | ACTCTTCACCTGCTCCACTG | GCTATGCTGCCTGCTCTTAC |
| Mouse iNOS | TCACCTTCGAGGGCAGCCGA | TCCGTGGCAAAGCGAGCCAG |
| Mouse Arg1 | CCAGATGTACCAGGATTCTC | AGCAGGTAGCTGAAGGTCTC |
| Mouse IL-1β | AGCTTCAGGCAGGCAGTATC | TCATCTCGGAGCCTGTAGTG |
| Mouse IL-6 | AAGTCCGGAGAGGAGACTTC | TGGATGGTCTTGGTCCTTAG |
| Mouse CD206 | CTGCAGATGGGTGGGTTATT | GGCATTGATGCTGCTGTTATG |
| Mouse TGFβ | CGGAGAGCCCTGGATACCA | GCCGCACACAGCAGTTCTT |
| Mouse GAPDH | AGAACATCATCCCTGCCTCTACT | GATGTCATCATATTTGGCAGGTT |

SUPPLEMENTAL Methods

**Cardiac function analysis**

Cardiac function was evaluated in vivo using Vevo®2100 echocardiography (Visualsonics, Toronto) prior to, and three days following I/R induction. After anesthesia induction using isoﬂurane gas, Two-dimensional short and long axes of hearts were observed and imaged in 2-D and M-mode under electrocardiographic monitoring of heart rate. Three representative cycles were captured from each animal at each time point. Measurements for interventricular septal end-systolic thickness (IVSs), left ventricular posterior wall end-systolic thickness (LVPWs), interventricular septal end-diastolic thickness (IVSd), left ventricular posterior wall end-diastolic thickness (LVPWd), left ventricular internal diameter end-systolic (LVIDs), left ventricular internal diameter end-diastolic (LVIDd) were obtained and averaged on the M-mode tracings. LV fractional shortening (FS%) was determined as [(LVID;d–LVID;s)/LVID;d]× 100. LV ejection fraction (EF%) was calculated as: EF (%) = ((LV Vol;d-LV Vol;s)/LV Vol;d) × 100. LV Vol;d = ((7.0 / (2.4 + LVID;d)) × LVID;d^3^); LV Vol;s = ((7.0 / (2.4 + LVID;s)) × LVID;s^3^).

**MVO size measurements**

According to our previous study[1], MVO was determined using Thioflavin S staining. Briefly, 4% Thiofavin S (Cat Number T1892, Sigma Aldrich) was injected via aorta into the reperfused myocardium 24 hours after operation. Then the hearts were quickly frozen at -80°C and sliced into 1-mm thick slices along the short axis. Slices were exposed to UV light (302 nm) and MVO area (attenuation or absence of thiofavin S fluorescence) were manually traced using ImageJ software. The percentage of MVO was determined by dividing the area of MVO by the total area of the left ventricle (LV) and multiplying by 100%.

**Immunohistochemistry and H&E staining**

After intraventricular injection of 10% potassium chloride (KCl), hearts were arrested in diastole, excised and washed in PBS. After fixation with 4% phosphate-buffered formalin (pH 7.4), tissues were dehydrated, embedded in paraffin, cut into 5μm sections, and stained with anti-CD68 primary antibody (Abcam, ab125212) or hematoxylin and eosin following the manufacturer's instructions. To analyze the infiltration of inflammatory cell, the percentage of inflammatory cells was calculated by counting the number of inflammatory cells cellular nuclei and comparing them with the total number of cellular nuclei in the same area according to our previous study[2].

**Immunofluorescence**

After euthanization, hearts were perfused with ice-cold PBS and immediately embedded in OCT compound at −80 °C. Tissue samples were cut at 5-μm thickness slices and fixed with paraformaldehyde. After permeabilization with 0.1% Triton X-100 and blocking with 1% BSA, slices were incubated with the following primary antibodies: iNOS (Abcam, ab178945), and Arginase-1 (Arg1; Cell Signaling, #93668). Nucleus was counterstained with DAPI (Sigma Aldrich) following the application of the appropriate fluorescently conjugated secondary antibodies (Invitrogen)

**Cell culture and experimental protocol**

RAW264.7, the mouse macrophage cell line, was acquired from the American Type Culture Collection and cultured in Dulbecco's Modified Eagle Medium (DMEM; Gibco) supplemented with 10% of fetal bovine serum (FBS; Gibco) and 1% Penicillin/Streptomycin (Hyclone). RAW264.7 cells were pre-cultured in serum-free culture medium for 12 h to reduce mitogenic effects. Appropriately 1.0 x 10^6^ RAW264.7 cells were placed in a 6 well plate containing 2ml of medium. RAW264.7 cells were then treated with 100 ng/ml lipopolysaccharide (LPS, Sigma Aldrich) for 4h to induce an inflammatory microenvironment and then culture medium was refreshed. For co-culture experiment, EAT of mice were cultured in a transwell system (0.4 μm) which was placed above the RAW264.7 cells. The macrophages and adipose tissues share the same culture medium without direct cell-cell contact. After 48h, the cells were collected for further analysis.

**Cytokine Analysis in Heart tissue**

Three days following myocardial I/R injury, heart tissues were collected and homogenized with an electric homogenizer. The protein levels of IL‐10, IL-1β and IL‐6 were detected using the corresponding ELISA kit following the guidelines provided by the manufacturer (MultiScience, Hangzhou, China).

**RNA isolation and quantitative RT-PCR**

Total RNA was extracted from heart tissues or cells using TRIzol reagent (Invitrogen) following the manufacturer’s instructions. The purity and quantity of extracted RNA were determined using a NanoDrop Spectrophotometer (Thermo Scientific). For gene expression evaluation, cDNA was synthesized from mRNA using the HiScript II 1st Strand cDNA Synthesis Kit (Vazyme). The resulting cDNA was then mixed with ChamQ SYBR qPCR Master Mix (Vazyme) and designated primer sets and subsequently run on Step‐one plus Real‐Time PCR System (Applied Biosystems, CA). The quantified data were normalized to GAPDH mRNA relative treated controls and analyzed using the 2^−ΔΔCt^ method. The sequences of the primers are shown in supplementary Table 1.

**Flow cytometry analysis**

Cardiac tissues single‐cell suspensions of cardiac tissues were obtained using gentleMACS™ Dissociator (Miltenyi Biotec, Bergisch Gladbach, Germany) according to methods described by Meeson A et al[3]. Initially, Samples were incubated with an Fc receptor block (CD16/32, BD Bioscience) to reduce nonspecific antibody binding and then stained with CD11b‐FITC (BD Bioscience) and F4/80-PerCP/Cy5.5 (eBioscience) for 20 to 30 minutes at 4°C. For intracellular staining, cells were fixed and permeabilized using the intracellular Fixation and Permeabilization kit (eBioscience) and then stained with iNOS-PE (eBioscience) and CD206-APC (eBioscience). Flow cytometry was conducted using FACS Aria flow cytometer (BD Bioscience) and data were processed using FlowJo software (TreeStar, Ashland, USA).

**Bulk mRNA sequence**

Total RNA was isolated from EAT as described previously[4] and the integrity and concentration of RNA samples were determined by RNA Nano 6000 Assay Kit using the Bioanalyzer 2100 system (Agilent Technologies, Germany). A total of 500ng RNA per sample was used as input material for the RNA sample preparations. The sequencing libraries were generated using the NEBNext® Ultra™ RNA Library Prep Kit for Illumina® (NEB, USA) and the index codes were added to assign the sequences to each sample. Final libraries were analyzed on a Bioanalyzer and sequenced using 150-bp paired-end reads on an Illumina platform on the Illumina HiSeq2500. We filtered low-quality reads from raw data to obtain high-quality clean reads, which were aligned to the mouse genome (GRCm39) using Hisat2 (v. 2.1.0). Feature Counts (version 1.5) was utilized to calculate the read numbers mapped to each gene. DESeq2 R package (1.20.0) was used for differential expression analysis. A fold change of more than 2 and an adjusted P value of less than 0.05 were considered thresholds for differentially expressed genes (DEGs). The clusterProfiler (v. 4.0.2) in the R package was used for Kyoto Encyclopedia of Genes and Genomes (KEGG) enrichment analysis of the DEGs. The FDR-adjusted P-value was adjusted using the Benjamini-Hochberg method. The sequencing data of our study can be found in the NCBI Sequence Read Archive under accession no. PRJNA1089954 after the indicated release date.

**SUPPLEMENTAL REFERENCES**

1. Zhao J, Zhang Q, Cheng W, Dai Q, Wei Z, Guo M, Chen F, Qiao S, Hu J, Wang J, et al. Heart-gut

microbiota communication determines the severity of cardiac injury after myocardial ischaemia/reperfusion. Cardiovasc Res. 2023;119(6):1390-1402.

2. Zhao J, Li X, Hu J, Chen F, Qiao S, Sun X, Gao L, Xie J, Xu B. Mesenchymal stromal cell-derived

exosomes attenuate myocardial ischaemia-reperfusion injury through miR-182-regulated macrophage

polarization. Cardiovasc Res. 2019;115(7):1205-1216.

3. Meeson A, Fuller A, Breault DT, Owens WA, Richardson GD. Optimised protocols for the

identification of the murine cardiac side population. Stem Cell Rev Rep. 2013;9(5):731-9.

4. Chechi K, Vijay J, Voisine P, Mathieu P, Bossé Y, Tchernof A, Grundberg E, Richard D. UCP1

expression-associated gene signatures of human epicardial adipose tissue. JCI Insight. 2019;4(8):e123618.
